# Efficacy and safety of palbociclib in combination with letrozole as first-line treatment of ER-positive, HER2-negative, advanced breast cancer: expanded analyses of subgroups from the randomized pivotal trial PALOMA-1/TRIO-18

**DOI:** 10.1186/s13058-016-0721-5

**Published:** 2016-06-28

**Authors:** Richard S. Finn, John P. Crown, Johannes Ettl, Marcus Schmidt, Igor M. Bondarenko, Istvan Lang, Tamas Pinter, Katalin Boer, Ravindranath Patel, Sophia Randolph, Sindy T. Kim, Xin Huang, Patrick Schnell, Sashi Nadanaciva, Cynthia Huang Bartlett, Dennis J. Slamon

**Affiliations:** Department of Medicine, Division of Hematology/Oncology, David Geffen School of Medicine at University of California Los Angeles, Los Angeles, CA USA; Irish Cooperative Oncology Research Group, Dublin, Ireland; Technical University of Munich, Munich, Germany; University Hospital Mainz, Mainz, Germany; Dnipropetrovsk Medical Academy, City Multiple-Discipline Clinical Hospital, Dnipropetrovsk, Ukraine; Orszagos Onkologiai Intezet, Budapest, Hungary; Petz Aladar Megyei Oktato Korhaz, Gyor, Hungary; Department of Medical Oncology, Szent Margit Korhaz, Onkologia, Budapest, Hungary; Comprehensive Blood and Cancer Center, Bakersfield, CA USA; Pfizer Oncology, La Jolla, CA USA; Pfizer Oncology, New York, NY USA; Pfizer Oncology, Groton, CT USA; Pfizer Oncology, Collegeville, PA USA

**Keywords:** Breast cancer, Estrogen receptor-positive, HER2-negative, Cyclin-dependent kinase, Neutropenia

## Abstract

**Background:**

Palbociclib is an oral small-molecule inhibitor of cyclin-dependent kinases 4 and 6. In the randomized, open-label, phase II PALOMA-1/TRIO-18 trial, palbociclib in combination with letrozole improved progression-free survival (PFS) compared with letrozole alone as first-line treatment of estrogen receptor (ER)-positive, human epidermal growth factor receptor 2 (HER2)-negative, advanced breast cancer (20.2 months versus 10.2 months; hazard ratio (HR) = 0.488, 95 % confidence interval (CI) 0.319–0.748; one-sided *p* = 0.0004). Grade 3–4 neutropenia was the most common adverse event (AE) in the palbociclib + letrozole arm. We now present efficacy and safety analyses based on several specific patient and tumor characteristics, and present in detail the clinical patterns of neutropenia observed in the palbociclib + letrozole arm of the overall safety population.

**Methods:**

Postmenopausal women (*n* = 165) with ER+, HER2-negative, advanced breast cancer who had not received any systemic treatment for their advanced disease were randomized 1:1 to receive either palbociclib in combination with letrozole or letrozole alone. Treatment continued until disease progression, unacceptable toxicity, consent withdrawal, or death. The primary endpoint was PFS. We now analyze the difference in PFS for the treatment populations by subgroups, including age, histological type, history of prior neoadjuvant/adjuvant systemic treatment, and sites of distant metastasis, using the Kaplan-Meier method. HR and 95 % CI are derived from a Cox proportional hazards regression model.

**Results:**

A clinically meaningful improvement in median PFS and clinical benefit response (CBR) rate was seen with palbociclib + letrozole in every subgroup evaluated. Grade 3–4 neutropenia was the most common AE with palbociclib + letrozole in all subgroups. Analysis of the frequency of neutropenia by grade during the first six cycles of treatment showed that there was a downward trend in Grade 3–4 neutropenia over time. Among those who experienced Grade 3–4 neutropenia, 71.7 % had no overlapping infections of any grade and none had overlapping Grade 3–4 infections.

**Conclusion:**

The magnitude of clinical benefit seen with the addition of palbociclib to letrozole in improving both median PFS and CBR rate is consistent in nearly all subgroups analyzed, and consistent with that seen in the overall study population. The safety profile of the combination treatment in all subgroups was also comparable to that in the overall safety population of the study.

**Electronic supplementary material:**

The online version of this article (doi:10.1186/s13058-016-0721-5) contains supplementary material, which is available to authorized users.

## Background

Cyclin-dependent kinases (CDKs) are a large family of serine-threonine kinases that play several critical roles in cell cycle regulation. They are an attractive therapeutic target given that alterations in cell cycle genes have been implicated in breast cancer as well as multiple other malignancies. Palbociclib (PD-0332991) is a first-in-class, orally active, selective, reversible inhibitor of CDK 4 and 6 [1]. Pre-clinical studies have demonstrated the activity of palbociclib in blocking growth of estrogen receptor (ER)-positive luminal cell lines alone and in combination with anti-estrogens [2]. Clinical data have recently established palbociclib as a novel therapeutic option for women with ER+, human epidermal growth factor receptor 2 (HER2)-negative, advanced breast cancer [3–5]. In the randomized, open-label, phase II PALOMA-1/TRIO-18 trial, the safety and efficacy of palbociclib in combination with the aromatase inhibitor letrozole was compared with letrozole alone as first-line treatment of women with ER-positive, HER2-negative (ER+/HER2–), advanced breast cancer [3]. A total of 165 postmenopausal women with ER+/HER2– advanced breast cancer who had not received any treatment for their advanced disease were randomized (1:1), 84 to palbociclib plus letrozole and 81 to letrozole alone. The study stratified patients by disease site and disease-free interval. The primary endpoint was investigator-assessed progression-free survival (PFS). At the time of the data cut-off for the final analysis (29 November 2013), median follow-up was 29.6 months (95 % confidence interval (CI) 27.9–36.0) for the palbociclib plus letrozole arm and 27.9 months (95 % CI 25.5–31.1) for the letrozole arm [3]. Median PFS in the intention-to-treat (ITT) population was 20.2 months (95 % CI 13.8–27.5) for the palbociclib plus letrozole arm and 10.2 months (95 % CI 5.7–12.6) for the letrozole arm (hazard ratio (HR) = 0.488, 95 % CI 0.319–0.748; one-sided *p* = 0.0004) [3]. The clinical benefit response (CBR) rate (complete response + partial response + stable disease ≥24 weeks) for the ITT population was 81 % in the palbociclib plus letrozole arm and 58 % in the letrozole arm [3]. Overall survival data were not mature at the time of the data cut-off. Grade 3 neutropenia was the most common adverse effect in the palbociclib plus letrozole arm (48 % versus 1 % in the letrozole alone arm) [3]. On the basis of these data, palbociclib (Ibrance®, Pfizer) was granted accelerated approval by the US Food and Drug Administration (FDA) in February 2015, in combination with letrozole as initial endocrine-based therapy for postmenopausal women with ER+/HER2– metastatic breast cancer [6].

Despite the high incidence of neutropenia in the palbociclib plus letrozole arm of the PALOMA-1/TRIO-18 trial, there were no reported cases of febrile neutropenia or neutropenia-related infections. This is in contrast to cytotoxic chemotherapy where neutropenia is not only a common dose-limiting toxicity but is also associated with serious infections and infection-related morbidity [7, 8]. Ensuing complications can result in hospitalization, high treatment costs, and increased mortality [9–11].

There are two objectives in the current study: 1) to perform an expanded analysis of the efficacy (PFS and CBR) and safety of palbociclib plus letrozole in clinically relevant subgroups from the PALOMA-1/TRIO-18 trial including age <65 years or ≥65 years, pathologic type (ductal versus lobular carcinoma), effect of prior neoadjuvant/adjuvant systemic treatment, and metastasis in bone only, visceral sites, or other sites (i.e., soft-tissue, nodes, and so forth); and 2) to further delineate clinical patterns of neutropenia associated with palbociclib in the PALOMA-1/TRIO-18 trial in order to aid clinicians in the management of palbociclib-induced neutropenia.

## Methods

### Study design

The PALOMA-1/TRIO-18 trial was an international, phase II, multicenter, open-label, randomized study. Details of the study have been reported previously [3]. In brief, postmenopausal women with ER+/HER2– advanced breast cancer were enrolled in two cohorts that accrued sequentially. Patients in both cohorts 1 and 2 were required to have advanced breast cancers defined as ER-positive determined by immunohistochemistry, and HER2-negative determined either by fluorescent in-situ hybridization (FISH) or immunohistochemistry in local laboratory testing. Patients in cohort 2 were additionally required to have cancers with amplification of cyclin D1 (*CCND1*), loss of p16 (INK4A or CDKN2A), or both, as determined in central laboratory testing. Amplification of *CCND1* and loss of p16 were determined by a four-color FISH assay as previously described [3]. Patients in both cohorts were randomized 1:1 to receive palbociclib plus letrozole or letrozole alone and were stratified by disease site (bone-only, visceral, other) and disease-free interval (>12 months from completion of adjuvant treatment to disease recurrence versus ≤12 months or de novo advanced disease). For the final analysis of the primary endpoint, a combined analysis of cohorts 1 and 2 was performed [3].

All patients provided informed consent before any study-specific screening procedures were performed. The study was conducted in accordance with the International Conference on Harmonization and guidelines on Good Clinical Practice and was approved by the institutional review boards of the participating centers. The study is registered with the ClinicalTrials.gov, number NCT00721409.

### Patients

Postmenopausal women (≥18 years) with ER+/HER2– advanced breast cancer with evidence of 1) locally recurrent disease not amenable to resection or radiation therapy with curative intent or 2) metastatic disease were eligible. Additional key inclusion criteria included measurable disease by Response Evaluation Criteria in Solid Tumors (RECIST, version 1.0) or bone-only disease, an Eastern Cooperative Oncology Group (ECOG) performance status of 0 or 1, and adequate organ (bone marrow, renal, and hepatic) function. Key exclusion criteria included previous treatment for advanced breast cancer, prior neoadjuvant treatment with letrozole with disease recurrence ≤12 months, previous treatment with a CDK inhibitor, or presence of brain metastasis.

### Study treatment

Patients randomized to the palbociclib plus letrozole arm (*n* = 84) received oral palbociclib 125 mg once daily for 3 weeks followed by 1 week off in 28-day cycles plus oral letrozole 2.5 mg once daily. Patients randomized to the letrozole arm (*n* = 81) received oral letrozole 2.5 mg once daily. Study treatment continued until disease progression, unacceptable toxicity, consent withdrawal, or death.

### Endpoints and assessments

The primary endpoint was investigator-assessed PFS, defined as the time from randomization to objective disease progression or death on study. Secondary efficacy endpoints were objective response (by RECIST version 1.0), CBR, duration of response, and overall survival (OS). Additional secondary endpoints were safety, tissue/serum biomarker analyses, and patient-reported outcomes.

Tumor assessments of the chest, abdomen, and pelvis were performed by computed tomography or magnetic resonance imaging and clinical assessment of superficial disease (if applicable) at screening and every 8 weeks thereafter. Bone lesions (when applicable) were assessed by radiography; bone scans were performed at baseline and every 12 weeks. Safety was monitored throughout the study and adverse events (AEs) were graded according to the National Cancer Institute Common Terminology Criteria for Adverse Events (CTCAE) version 3.0. Hematology and blood chemistry assessments were done every 2 weeks for the first two treatment cycles and at the beginning of each subsequent cycle. Dosing modifications were performed as described in Additional file 1 (Table S1). Primary prophylactic use of granulocyte colony-stimulating factors was not permitted, although these were allowed to treat treatment-emergent neutropenia as indicated by current ASCO guidelines. Erythropoietin was permitted at the investigator’s discretion for the supportive treatment of anemia.

### Statistical analysis

Statistical analysis of the overall study population was previously described in detail [3]. All efficacy analyses were performed on the ITT population; safety analyses included all patients who had received at least one dose of the study treatment. PFS was assessed using the Kaplan-Meier method; HR and 95 % CI were derived from a Cox proportional hazards regression model. No adjustments were made for multiple comparisons in the subgroup analyses since they are considered exploratory analyses. All statistical analyses were performed with SAS version 9.2 or later.

## Results

### Subgroup analyses: baseline characteristics

The baseline characteristics of the subgroups are summarized in Table 1. In the ITT population (*n* = 165), 54 % of patients were <65 years of age. The majority (71 %) of patients had ductal carcinoma and 22.4 % had lobular carcinoma (the remaining 6.6 % of patients had mixed histology, tubular, or mucinous types). Approximately half (49 %) of the patients had not received prior neoadjuvant or adjuvant systemic treatment. Twenty-nine (17.6 %) patients had bone-only disease at baseline, 48.5 % of patients had visceral metastases (lung and/or liver ± any other site), and 33.9 % of patients had other metastases (bone with other non-visceral sites or other disease sites alone). In the subgroup that had visceral metastases, 52.5 % had metastases in the liver and 76 % had metastases in the lungs; further details on the baseline characteristics of the patients within this subgroup are summarized in Additional file 1 (Table S2). In general, baseline clinical characteristics in the palbociclib plus letrozole arm and letrozole alone arm were well balanced within each subgroup (Table 1), although there were slight imbalances in disease site and prior systemic treatment.

**Table 1:** Baseline characteristics of subgroups (intention-to-treat population)

Age group	<65 years (*n* = 89)			≥65 years (*n* = 76)		
	P+ L (*n* = 47)	L (*n* = 42)		P + L (*n* = 37)	L (*n* = 39)	
Median age (range), years	55.0 (41–64)	56.5 (38–64)		72.0 (65–89)	70.0 (65–84)	
ECOG performance status						
0	26 (55.3 %)	23 (54.8 %)		20 (54.1 %)	22 (56.4 %)	
1	21 (44.7 %)	19 (45.2 %)		17 (45.9 %)	17 (43.6 %)	
Disease stage						
IIIB	1 (2.1 %)	1 (2.4 %)		1 (2.7 %)	0	
IV	46 (97.9 %)	41 (97.6 %)		36 (97.3 %)	39 (100.0 %)	
Disease site^a^						
Bone-only	9 (19.2 %)	3 (7.1 %)		8 (21.6 %)	9 (23.1 %)	
Visceral	19 (40.4 %)	22 (52.4 %)		18 (48.7 %)	21 (53.9 %)	
Other	19 (40.4 %)	17 (40.5 %)		11 (29.7 %)	9 (23.1 %)	
Prior systemic treatment						
None	24 (51.1 %)	23 (54.8 %)		20 (54.1 %)	14 (35.9 %)	
Yes	23 (48.9 %)	19 (45.2 %)		17 (45.9 %)	25 (64.1 %)	
Histological type	Ductal carcinoma (*n* = 117)			Lobular carcinoma (*n* = 37)		
	P+ L (*n* = 63)	L (*n* = 54)		P + L (*n* = 18)	L (*n* = 19)	
Median age (range), years	63 (41–89)	65 (42–84)		60 (50–74)	62 (38–78)	
ECOG performance status						
0	36 (57.1 %)	29 (53.7 %)		9 (50.0 %)	14 (73.7 %)	
1	27 (42.9 %)	25 (46.3 %)		9 (50.0 %)	5 (26.3 %)	
Disease stage						
IIIB	2 (3.2 %)	1 (1.9 %)		0	0	
IV	61 (96.8 %)	53 (98.1 %)		18 (100.0 %)	19 (100.0 %)	
Disease site^a^						
Bone-only	11 (17.5 %)	8 (14.8 %)		4(22.2 %)	3 (15.8 %)	
Visceral	28 (44.4 %)	30 (55.6 %)		8 (44.4 %)	7 (36.8 %)	
Other	24 (38.1 %)	16 (29.6 %)		6 (33.3 %)	9 (47.4 %)	
Prior systemic treatment						
None	36 (57.1 %)	26 (48.1 %)		6 (33.3 %)	7 (36.8 %)	
Yes	27 (42.9 %)	28 (51.9 %)		12 (66.7 %)	12 (63.2 %)	
Prior systemic treatment	None (*n* = 81)			Yes (n = 84)		
	P+ L (*n* = 44)	L (*n* = 37)		P + L (*n* = 40)	L (*n* = 44)	
Median age (range), years	63.0 (41–83)	62.0 (43–76)		60.0 (46–89)	65.5 (38–84)	
ECOG performance status						
0	26 (59.1 %)	18 (48.6 %)		20 (50.0 %)	27 (61.4 %)	
1	18 (40.9 %)	19 (51.4 %)		20 (50.0 %)	17 (38.6 %)	
Disease stage						
IIIB	1 (2.3 %)	0		1 (2.5 %)	1 (2.3 %)	
IV	43 (97.7 %)	37 (100.0 %)		39 (97.5 %)	43 (97.7 %)	
Disease site^a^						
Bone-only	6 (13.6 %)	5 (13.5 %)		11 (27.5 %)	7 (15.9 %)	
Visceral	24 (54.6 %)	19 (51.4 %)		13 (32.5 %)	24 (54.6 %)	
Other	14 (31.8 %)	13 (35.1 %)		16 (40.0 %)	13 (29.6 %)	
Disease site^a^	Bone-only (*n* = 29)		Visceral (*n* = 80)		Other (*n* = 56)	
	P + L (*n* = 17)	L (*n* = 12)	P + L (*n* = 37)	L (*n* = 43)	P + L (*n* = 30)	L (*n* = 26)
Median age (range), years	63.0 (46–89)	69.0 (42–73)	63.0 (41–83)	64.0 (38–84)	62.0 (52–78)	62.0 (43–75)
ECOG performance status						
0	11 (64.7 %)	10 (83.3 %)	19 (51.4 %)	19 (44.2 %)	16 (53.3 %)	16 (61.5 %)
1	6 (35.3 %)	2 (16.7 %)	18 (48.6 %)	24 (55.8 %)	14 (46.7 %)	10 (38.5 %)
Disease stage						
IIIB	0	0	0	0	2 (6.7 %)	1 (3.8 %)
IV	17 (100.0 %)	12 (100.0 %)	37 (100.0 %)	43 (100.0 %)	28 (93.3)	25 (96.2)
Prior systemic treatment						
None	6 (35.3 %)	5 (41.7 %)	24 (64.9 %)	19 (44.2 %)	14 (46.7 %)	13 (50.0 %)
Yes	11 (64.7 %)	7 (58.3 %)	13 (35.1 %)	24 (55.8 %)	16 (53.3 %)	13 (50.0 %)

Data are n (%) unless otherwise indicated

^a^Based on case report form data

*ECOG* Eastern Cooperative Oncology Group, *L* letrozole alone, *P + L* palbociclib + letrozole

### Subgroup analyses: efficacy

**Fig. 1:**
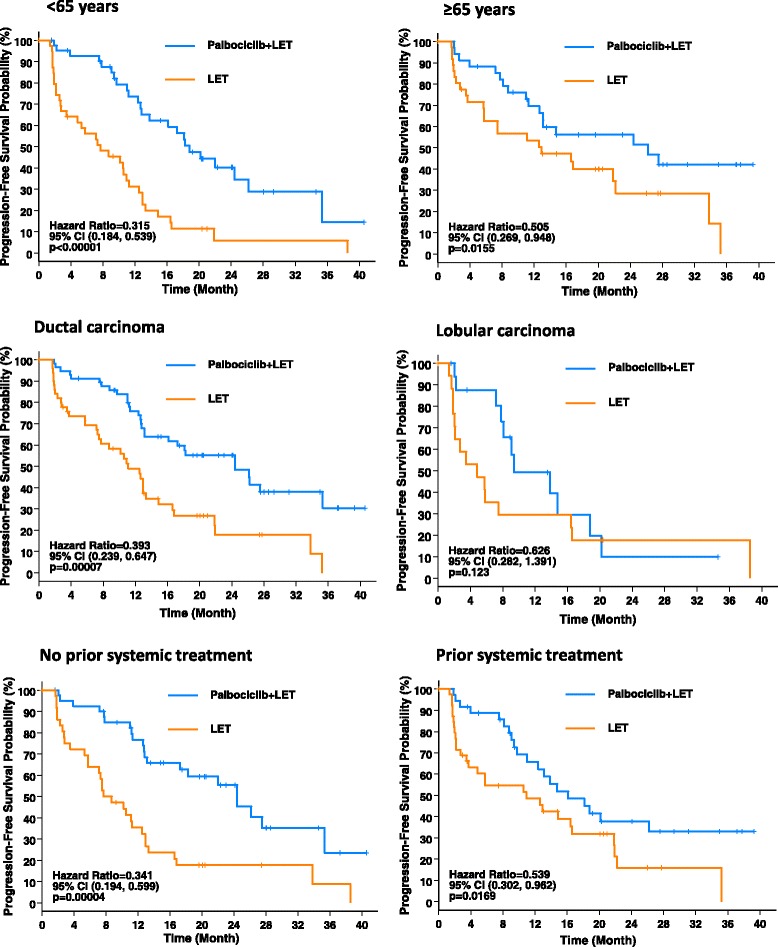
Progression-free survival (intention-to-treat population) in subgroups based on age, histological type of breast cancer, and history of prior neoadjuvant/adjuvant systemic treatment. *CI* confidence interval, *LET* letrozole

**Fig. 2:**
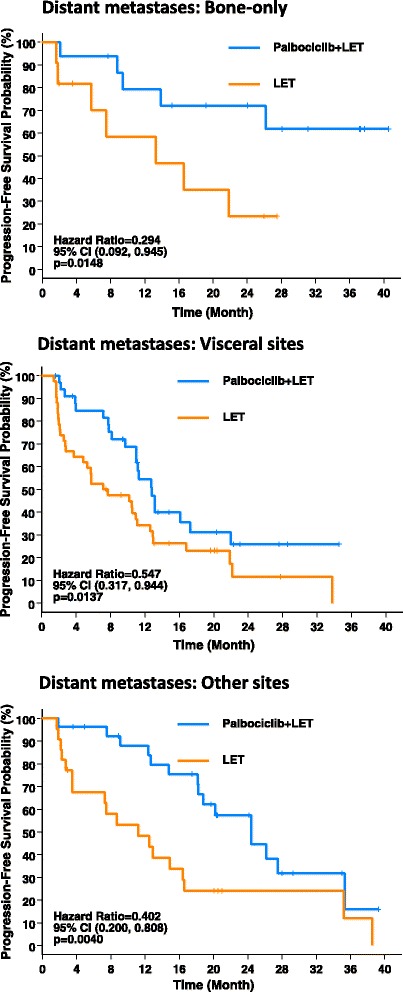
Progression-free survival (intention-to-treat population) in subgroups based by distant metastases. *CI* confidence interval, *LET* letrozole

The median PFS and the CBR rate for the subgroups are summarized in Table 2; Kaplan-Meier plots of PFS for the subgroups were determined (Figs. 1 and 2).

**Table 2:** Progression-free survival (PFS) and clinical benefit response (CBR) rate of subgroups (intention-to-treat population)

Age group	<65 years (*n* = 89)			≥65 years (*n* = 76)		
	P+ L (*n* = 47)		L (*n* = 42)	P + L (*n* = 37)		L (*n* = 39)
Median PFS (95 % CI), months	18.8 (12.8–26.1)		7.7 (2.8–10.9)	26.2 (12.6–NE)		12.9 (5.7–22.2)
HR (95 % CI)	0.315 (0.184–0.539)			0.505 (0.269–0.948)		
CBR* rate (95 % CI), %	80.9 (66.7–90.9)		54.8 (38.7–70.2)	81.1 (64.8–92.0)		61.5 (44.6–76.6)
Histological type	Ductal carcinoma (*n* = 117)			Lobular carcinoma (*n* = 37)		
	P+ L (*n* = 63)		L (*n* = 54)	P + L (*n* = 18)		L (*n* = 19)
Median PFS (95 % CI), months	24.4 (13.1–35.3)		11.1 (7.3–13.3)	9.4 (7.8–18.8)		4.8 (1.9–16.4)
HR (95 % CI)	0.393 (0.239–0.647)			0.626 (0.282–1.391)		
CBR* rate (95 % CI), %	82.5 (70.9–90.9)		63.0 (48.7–75.7)	72.2 (46.5–90.3)		42.1 (20.3–66.5)
Prior systemic treatment	None (*n* = 81)			Yes (*n* = 84)		
	P + L (*n* = 44)		L (*n* = 37)	P + L (*n* = 40)		L (*n* = 44)
Median PFS (95 % CI), months	24.4 (13.1–35.3)		8.2 (5.7–12.5)	16.1 (11–NE)		10.9 (3.5–16.6)
HR (95 % CI)	0.341 (0.194–0.599)			0.539 (0.302–0.962)		
CBR* rate (95 % CI), %	84.1 (69.9–93.4)		70.3 (53.0–84.1)	77.5 (61.5–89.2)		47.7 (32.5–63.3)
				Prior anti-hormone treatment (n = 55)		
				P + L (n = 27)		L (n = 28)
Median PFS (95 % CI), months	NA			18.8 (9.7–NE)		12.9 (2.1–21.8)
HR (95 % CI)	NA			0.460 (0.222–0.956)		
CBR* rate (95 % CI), %	NA			77.8 (57.7–91.4)		53.6 (33.9–72.5)
Disease site^a^	Bone-only (*n* = 29)		Visceral (*n* = 80)		Other (*n* = 56)	
	P + L (*n* = 17)	L (*n* = 12)	P + L (*n* = 37)	L (*n* = 43)	P + L (*n* = 30)	L (*n* = 26)
Median PFS (95 % CI), months	NE (9.4–NE)	13.3 (1.8–NE)	12.8 (9.7–17.2)	7.4 (3.7–11.1)	24.4 (18.1–35.3)	11.2 (3.5–16.4)
HR (95 % CI)	0.294 (0.092–0.945)		0.547 (0.317–0.944)		0.402 (0.200–0.808)	
CBR* rate (95 % CI), %	88.2 (63.6–98.5)	58.3 (27.7–84.8)	75.5 (58.5–88.2)	60.5 (44.4–75.0)	83.3 (65.3–94.4)	53.8 (33.4–73.4)

*The CBR was determined by investigator assessment

^a^Based on case report form data

*CI* confidence interval, *HR* hazard ratio, *L* letrozole alone, *NA* not applicable, *NE* not estimable, *P + L* palbociclib + letrozole

Palbociclib plus letrozole improved median PFS in both subgroups of age. In patients <65 years of age, median PFS was 18.8 months (95 % CI 12.8–26.1) with palbociclib plus letrozole and 7.7 months (95 % CI 2.8–10.9) with letrozole alone (HR = 0.315, 95 % CI 0.184–0.539; *p* < 0.00001). In patients ≥65 years of age, median PFS was 26.2 months (95 % CI 12.6 to not estimable) with palbociclib plus letrozole and 12.9 months (95 % CI 5.7–22.2) with letrozole alone (HR = 0.505, 95 % CI 0.269–0.948; *p* = 0.0155).

Palbociclib plus letrozole improved median PFS in patients with ductal carcinoma as well as in patients with lobular carcinoma. Median PFS in the ductal population was 24.4 months (95 % CI 13.1–35.3) with palbociclib plus letrozole and 11.1 months (95 % CI 7.3–13.3) with letrozole alone (HR = 0.393, 95 % CI 0.239–0.647; *p* = 0.00007). The subgroup with lobular carcinoma was limited by the small patient numbers in each arm (*n* = 18 in the palbociclib plus letrozole arm; *n* = 19 in the letrozole alone arm). Median PFS in this subgroup was 9.4 months (95 % CI 7.8–18.8) with palbociclib plus letrozole and 4.8 months (95 % CI 1.9–16.4) with letrozole alone (HR = 0.626, 95 % CI 0.282–1.391; *p* = 0.123).

Palbociclib plus letrozole improved median PFS regardless of whether patients had received prior neoadjuvant/adjuvant systemic treatment. Median PFS in patients without prior neoadjuvant/adjuvant systemic treatment was 24.4 months (95 % CI 13.1–35.3) with palbociclib plus letrozole and 8.2 months (95 % CI 5.7–12.5) with letrozole alone (HR = 0.341, 95 % CI 0.194–0.599; *p* = 0.00004). Median PFS in patients with prior systemic treatment was 16.1 months (95 % CI 11 to not estimable) with palbociclib plus letrozole and 10.9 months (95 % CI 3.5–16.6) with letrozole alone (HR = 0.539, 95 % CI 0.302–0.962; *p* = 0.0169). Within the subgroup that had prior systemic treatment, 65.5 % of patients had prior anti-hormone treatment. Median PFS in patients with prior anti-hormone treatment was 18.8 months (95 % CI 9.7 to not estimable) with palbociclib plus letrozole and 12.9 months (95 % CI 2.1–21.8) with letrozole alone (HR = 0.460, 95 % CI 0.222–0.956; *p* = 0.0165).

The subgroup of patients with bone-only disease was limited by a small number of patients in each arm (*n* = 17 in the palbociclib plus letrozole arm; *n* = 12 in the letrozole alone arm). Despite this, palbociclib plus letrozole improved the median PFS compared with letrozole alone (Table 2). Median PFS in patients with either visceral metastases or distant metastases at other sites was higher with palbociclib plus letrozole than with letrozole alone (Table 2).

Palbociclib plus letrozole improved the CBR rate in all subgroups compared with letrozole alone (Table 2); the difference in the CBR rate with the combination treatment for each subgroup was consistent with that seen for the overall study population.

### Subgroup analyses: safety

The safety analyses on the subgroups are summarized in Table 3. The most common all-cause AEs that occurred in at least 10 % of patients in the palbociclib plus letrozole arm of the safety population for each subgroup are summarized in detail in Additional file 1 (Tables S3–S11).

**Table 3:** Safety analysis of subgroups (as treated population)

Age group	<65 years (*n* = 86)		≥65 years (*n* = 74)			
	P+ L (*n* = 46)	L (*n* = 40)	P + L (*n* = 37)		L (*n* = 37)	
AEs (all causality), n (%)	46 (100.0 %)	33 (82.5 %)	37 (100.0 %)		32 (86.5 %)	
Grade 3–4 AEs (all causality), n (%)	37 (80.4 %)	4 (10.0 %)	27 (73.0 %)		12 (32.4 %)	
5 most common AEs that had a higher (>10 %) incidence in the P + L arm than in the L arm	Neutropenia, leukopenia, fatigue, anemia, alopecia		Neutropenia, leukopenia, fatigue, anemia, nausea			
SAEs (all causality), n (%)	7 (15.2 %)	1 (2.5 %)	11 (29.7 %)		4 (10.8 %)	
Permanent discontinuation due to AEs (all causality), n (%)	6 (13.0 %)	1 (2.5 %)	6 (16.2 %)		1 (2.7 %)	
Dose reductions due to AEs (all causality), n (%)	18 (39.1 %)	NA	14 (37.8 %)		NA	
Histological type	Ductal carcinoma (*n* = 113)		Lobular carcinoma (*n* = 36)			
	P+ L (*n* = 62)	L (*n* = 51)	P + L (*n* = 18)		L (*n* = 18)	
AEs (all causality), n (%)	62 (100.0 %)	44 (86.3 %)	18 (100.0 %)		13 (72.2 %)	
Grade 3–4 AEs (all causality), n (%)	47 (75.8 %)	13 (25.5 %)	14 (77.8 %)		2 (11.1 %)	
5 most common AEs that had a higher (>10 %) incidence in the P + L arm than in the L arm	Neutropenia, fatigue, leukopenia, anemia, nausea		Neutropenia, anemia, leukopenia, asthenia, alopecia			
SAEs (all causality), n (%)	12 (19.4 %)	5 (9.8 %)	4 (22.2 %)		0	
Permanent discontinuation due to AEs (all causality), n (%)	8 (12.9 %)	1 (2.0 %)	4 (22.2 %)		1 (5.6 %)	
Dose reductions due to AEs (all causality), n (%)	27 (43.5 %)	NA	5 (27.8 %)		NA	
Prior systemic treatment	None (*n* = 80)		Yes (*n* = 80)			
	P+ L (*n* = 43)	L (*n* = 37)	P + L (*n* = 40)		L (*n* = 40)	
AEs (all causality), n (%)	43 (100.0 %)	32 (86.5 %)	40 (100.0 %)		33 (82.5 %)	
Grade 3–4 AEs (all causality), n (%)	30 (69.8 %)	5 (13.5 %)	34 (85.0 %)		11 (27.5 %)	
5 most common AEs that had a higher (>10 %) incidence in the P + L arm than in the L arm	Neutropenia, fatigue, leukopenia, anemia, alopecia		Neutropenia, leukopenia, anemia, nausea, thrombocytopenia			
SAEs (all causality), n (%)	7 (16.3 %)	2 (5.4 %)	11 (27.5 %)		3 (7.5 %)	
Permanent discontinuation due to AEs (all causality), n (%)	7 (16.3 %)	1 (2.7 %)	5 (12.5 %)		1 (2.5 %)	
Dose reductions due to AEs (all causality), n (%)	17 (39.5 %)	NA	15 (37.5 %)		NA	
Disease site^a^	Bone-only (*n* = 28)		Visceral (*n* = 80)		Other (*n* = 52)	
	P + L (*n* = 17)	L (*n* = 11)	P + L (*n* = 37)	L (*n* = 43)	P + L (*n* = 29)	L (*n* = 23)
AEs (all causality), n (%)	17 (100.0 %)	10 (90.9 %)	37 100.0 %)	36 (83.7 %)	29 (100.0 %)	19 (82.6 %)
Grade 3–4 AEs (all causality), n (%)	15 (88.2 %)	2 (18.2 %)	27 (73.0 %)	12 (27.9 %)	22 (75.8 %)	2 (8.7 %)
5 most common AEs that had a higher (>10 %) incidence in the P + L arm than in the L arm	Neutropenia, fatigue, leukopenia, nausea, anemia		Neutropenia, leukopenia, fatigue, anemia, decreased appetite		Neutropenia, leukopenia, anemia fatigue, hot flush	
SAEs (all causality), n (%)	4 (23.5 %)	1 (9.1 %)	10 (27.0 %)	3 (7.0 %)	4 (13.8 %)	1 (4.3 %)
Permanent discontinuation due to AEs (all causality), n (%)	2 (11.8 %)	0	7 (18.9 %)	1 (2.3 %)	3 (10.3 %)	1 (4.3 %)
Dose reductions due to AEs (all causality), n (%)	5 (29.4 %)	NA	14 (37.8 %)	NA	13 (44.8 %)	NA

^a^Based on case report form data

*AE* adverse event, *L* letrozole alone, *NA* not applicable, *P + L* palbociclib + letrozole, *SAE* serious adverse event

All subgroups had a higher incidence of Grade 3–4 AEs in the palbociclib plus letrozole arm than in the letrozole arm (≤88.2 % versus ≤32.4 %) (Table 3). There was one Grade 5 AE in the palbociclib plus letrozole arm: the event was death due to disease progression in a patient <65 years of age with lobular carcinoma and distant metastases at visceral sites who had not received prior systemic treatment.

Neutropenia, leukopenia, fatigue, and anemia were the most common AEs in all subgroups in the palbociclib plus letrozole study arm (Table 3). The incidence of all-causality Grade 3–4 AEs with palbociclib plus letrozole was generally similar in all subgroups, and so was the incidence of all-causality serious adverse events (SAEs) with the combination treatment.

The percentage of patients who had treatment discontinuations due to all-causality AEs with palbociclib plus letrozole was generally similar for all subgroups (Table 3). A similar trend was seen with the percentage of patients who had dose reductions due to all-causality AEs with palbociclib plus letrozole.

### Clinical patterns of neutropenia associated with palbociclib

Neutropenia was the most common AE in the palbociclib plus letrozole arm of the overall safety population of the study [3]. The key findings from the clinical patterns of neutropenia observed with palbociclib in the overall safety population are summarized in Table 4.

**Table 4:** Neutropenia clinical patterns in the overall safety population (as treated population) of the PALOMA-1/TRIO-18 trial

	Palbociclib + letrozole (*n* = 83)	Letrozole (*n* = 77)
Patients with neutropenia*, n (%)^a^		
All Grades	63 (75.9 %)	4 (5.2 %)
Grade 3	41 (49.4 %)	1 (1.3 %)
Grade 4	5 (6.0 %)	0
Laboratory abnormality: neutrophils, n (%)^‡^		
All Grades	77 (93.9 %)	13 (16.9 %)
Grade 3	47 (57.3 %)	2 (2.6 %)
Grade 4	4 (4.9 %)	0
Neutropenia episodes per patient, n (%)^a^		
1	10 (12.1 %)	2 (2.6 %)
2	9 (10.8 %)	1 (1.3 %)
3–5	15 (18.1 %)	0
≥ 6	29 (34.9 %)	1 (1.3 %)
Median time (range) from first dose to first episode of neutropenia onset, days		
Any grade	20.0 (13–757)	49.5 (15–113)
Grade ≥3	28.0 (14–757)	225.0 (225–225)
Grade 4	16.0 (14–246)	–
Total number of episodes of neutropenia in the study		
All Grades	472	16
Grade ≥3	265	4
Grade 4	11	0
Median duration (range) of neutropenia by episode, days		
Grade ≥3	8 (2–58)	30 (27–31)
Grade 4	7 (3–16)	–
Dose reductions, dose interruptions, or cycle delays due to any grade neutropenia, n (%)^a^	43 (51.8 %)	–
Permanent discontinuation from the study due to Grade 3–4 neutropenia, n (%)^a^	5 (6.0 %)	0
All Grades neutropenia with overlapping all Grades infections, n (%)^b^		
Yes	23 (36.5 %)	0
No	40 (63.5 %)	4 (100.0 %)
All Grades neutropenia with overlapping Grade 3–4 infections, n (%)^b^		
Yes	1 (1.6 %)	0
No	62 (98.4 %)	4 (100.0 %)
Grade 3–4 neutropenia with overlapping all Grades infections, n (%)^c^		
Yes	13 (28.2 %)	0
No	33 (71.7 %)	1 (100.0 %)
Grade 3–4 neutropenia with overlapping Grade 3–4 infections, n (%)^c^		
Yes	0	0
No	46 (100.0 %)	1 (100.0 %)

*Neutropenia included preferred terms “neutropenia” and “neutrophil count decreased” (MeDRA 16.1 coding dictionary)

^‡^The number of patients who had at least one on-study assessment for absolute neutrophil count was 82 in the palbociclib plus letrozole arm and 77 in the letrozole arm

^a^Percentages are based on the number of patients in each arm (*n*) of the study: 83 in the palbociclib plus letrozole arm, 77 in the letrozole arm

^b^Percentages are based on the number of patients with all Grades neutropenia: 63 in the palbociclib plus letrozole arm, 4 in the letrozole arm

^c^Percentages are based on the number of patients with Grade 3–4 neutropenia: 46 in the palbociclib plus letrozole arm, 1 in the letrozole arm

**Fig. 3:**
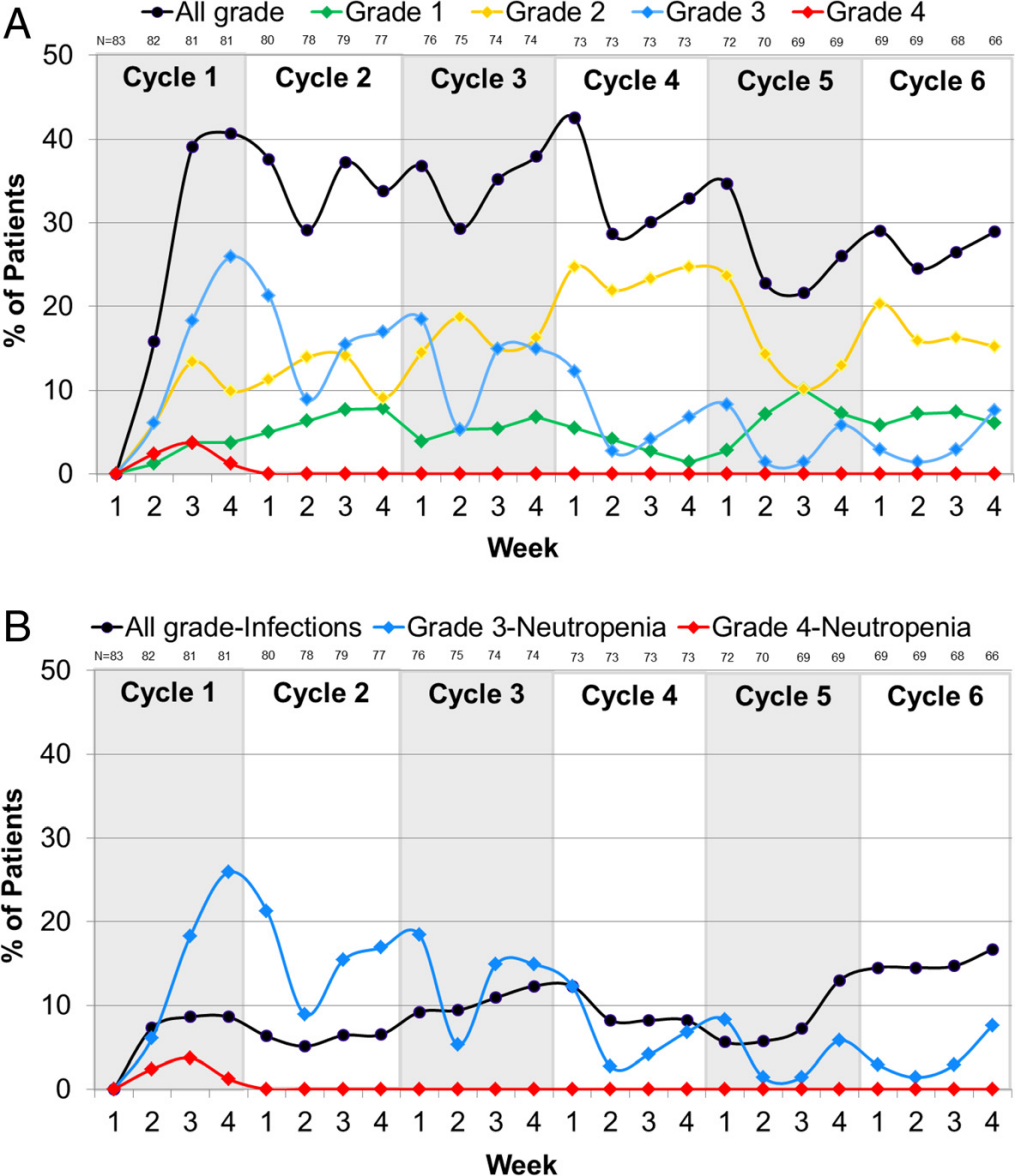
**a** Frequency of neutropenia by grade during the first six cycles of treatment in the palbociclib plus letrozole arm of the study. **b** Frequency of Grade 3–4 neutropenia and all Grade infections during the first six cycles of treatment in the palbociclib plus letrozole arm of the study

In the palbociclib plus letrozole arm, 75.9 % of patients had any grade neutropenia, 49.4 % had Grade 3 neutropenia, and 6.0 % had Grade 4 neutropenia (Table 4). In contrast, in the letrozole alone arm, 5.2 % of patients had any grade neutropenia, 1.3 % had Grade 3 neutropenia and no patient had Grade 4 neutropenia. The investigator-reported neutropenia AEs generally reflected neutrophil laboratory findings (Table 4). Approximately half (53 %) of the patients in the palbociclib plus letrozole arm had ≥3 episodes of neutropenia. The median time from first dose to first episode of any grade neutropenia in the palbociclib plus letrozole arm was 20 days. The median duration of Grade ≥3 neutropenia was 8 days and the median duration of Grade 4 neutropenia was 7 days. Approximately half (51.8 %) of the patients who had any grade neutropenia had dose reductions, dose interruptions, or cycle delays in the palbociclib plus letrozole arm but only five patients (6 %) were required to permanently discontinue treatment due to Grade 3–4 neutropenia as per protocol. When the frequency of neutropenia by grade during the first six cycles of treatment was analyzed (Fig. 3a), there was a downward trend in Grade 3–4 neutropenia over time, suggesting that there was no cumulative toxicity and that early dose modifications were likely effective in reducing the frequency of these events.

Since neutropenia usually increases the risk for infection, we explored the frequency of neutropenia with overlapping infections in the palbociclib plus letrozole arm. Only one patient with any grade neutropenia had an overlapping Grade 3–4 infection (Grade 3 influenza) in the palbociclib plus letrozole arm (Table 4). The majority (71.7 %) of patients with Grade 3–4 neutropenia in the palbociclib plus letrozole arm had no overlapping infections of any grade, and those that did had grade 1–2 infections only. When evaluating the first six cycles of treatment, there was no clear association between the frequency of Grade 3–4 neutropenia and all Grade infections (Fig. 3b). Furthermore, no patients with Grade 3–4 neutropenia had overlapping Grade 3–4 infections in the palbociclib plus letrozole arm (Table 4), nor were there any cases of febrile neutropenia in the study.

## Discussion

Hormone receptor-positive (HR+) breast cancer remains the most common sub-type of breast cancer. Historically, the most important therapeutic intervention in the management of patients with HR+ breast cancer has been the use of anti-estrogen therapy, including tamoxifen, aromatase inhibitors, and fulvestrant. Advanced breast cancer remains incurable, although with currently available therapies median survival is in the order of 3–4 years [12–14]. The PALOMA-1/TRIO-18 data and the regulatory approval of palbociclib represent the first novel non-endocrine therapy to improve PFS in the front-line setting of postmenopausal women with ER+/HER2– advanced breast cancer. While confirmation of this finding is pending the results of the PALOMA-2/TRIO-22 study which is a randomized, double-blind study of palbociclib and letrozole versus placebo and letrozole, results from the double-blind, placebo-controlled PALOMA-3 study, evaluating palbociclib and fulvestrant versus fulvestrant in a hormone-refractory population confirm the important role of CDK 4/6 inhibition in ER+/HER2– breast cancer. At this time, survival data from both studies are not mature, but the significant improvement in PFS of over 10 months in PALOMA-1/TRIO-18 is clinically meaningful. It is also interesting to note that the FIRST study, an open label, randomized, phase II study comparing fulvestrant versus anastrozole in a front-line, aromatase inhibitor-naive, ER+ population, demonstrated an improvement of 10 months in time to progression with fulvestrant (23.4 months for fulvestrant versus 13.1 months with anastrozole) [15] that ultimately resulted in an improvement in overall survival [12].

In the current study, we explored the clinical benefit and safety profile of palbociclib and letrozole in various sub-groups from the PALOMA-1/TRIO-18 trial, on the basis of both patient and tumor characteristics. When the subgroups were analyzed on the basis of age (<65 years and ≥65 years), there was a consistent benefit from the addition of palbociclib regardless of age. These observations are important given that age-related disparities have been reported for breast cancer outcomes [16]. Furthermore, the incidence of Grade 3/4 adverse events, the rate of dose reductions, and the rate of discontinuations were similar in the two age groups.

PALOMA-1/TRIO-18 was a front-line treatment study for advanced breast cancer, but about half of the patients had prior systemic therapy in the neoadjuvant/adjuvant setting. The prior systemic therapy received included both chemotherapy and anti-hormone approaches such as non-steroidal aromatase inhibitors, steroidal aromatase inhibitors, and tamoxifen. Consistent with our understanding of drug resistance, the largest benefit with palbociclib plus letrozole was seen in those patients that had never received any prior adjuvant therapy (HR = 0.341, 95 % CI 0.194–0.599; *p* = 0.00004). However, even in those patients that received prior systemic therapy, the benefit of treatment was clinically meaningful (HR = 0.539, 95 % CI 0.302–0.962; *p* = 0.0169). Importantly, in those patients that had had prior endocrine therapy, the HR was 0.46 (95 % CI 0.222–0.956; *p* = 0.0165), supporting our hypothesis that CDK 4/6 inhibition with palbociclib can overcome resistance to endocrine therapy [2].

In regard to tumor characteristics, we evaluated both histologic features and anatomic features. While the majority of invasive breast cancers are ductal in origin, approximately 10–15 % are lobular. As expected, the majority of patients in the PALOMA-1 trial had ductal carcinoma. In general, it is felt that lobular carcinomas have a better prognosis than ductal carcinomas, are more sensitive to endocrine therapy, and do not respond as well to systemic chemotherapy [17]. With these factors in mind, we explored whether there was a differential effect seen with the addition of palbociclib in these two histologies. When comparing the letrozole arms of the two subgroups, the ductal carcinoma subgroup had a better PFS than the lobular carcinoma subgroup (11.1 months versus 4.8 months). This observation clearly needs to be interpreted with caution given that there were only 19 patients in the letrozole arm of the lobular carcinoma subgroup and other clinical factors were not accounted for; for example, more patients with lobular carcinoma received adjuvant systemic therapy versus the ductal group. Nevertheless, both groups appear to have benefited from the addition of palbociclib, with a HR of 0.393 (95 % CI 0.239–0.647; *p* = 0.00007) in the ductal group and 0.626 (95 % CI 0.282–1.391; *p* = 0.123) in the lobular group. The *p* value of 0.123 in the lobular group likely reflects the small sample size. Whether or not this difference in degree of benefit is significant will need to be established in larger studies.

Another relevant baseline prognostic factor we evaluated was anatomic stage of the disease. While virtually all of the patients had Stage IV disease, there was a mixture of those that had visceral disease versus bone-only or other sites of disease, such as soft tissue or lymph node metastases. The HR values for these groups (visceral disease, bone-only, other sites) were 0.547 (95 % CI 0.317–0.944; *p* = 0.0137), 0.294 (95 % CI, 0.092–0.945; *p* = 0.0148), and 0.402 (95 % CI, 0.200–0.808; *p* = 0.0040), respectively. While the degree of benefit mimics the prognosis for these groups, even in the worst prognosis patient group (i.e., those with visceral disease) there was a 45 % decrease in the risk of progression with palbociclib. In addition, the clinical benefit rate in this group, while less than the other two groups, was still over 75 %, confirming that the combination is an appropriate option for women with visceral disease. This is particularly relevant given that this group of patients is often considered for front-line chemotherapy over endocrine therapy.

Finally, the therapeutic benefit of any new agent needs to be weighed against its side effects profile. As previously reported [3], the most common side effects with the combination therapy were neutropenia, leukopenia, and fatigue. Palbociclib is dosed using a 3-week on, 1-week off regimen to manage the neutropenia/leukopenia side effect. While the high incidence of Grade 3/4 events in the neutropenia/leukopenia categories is cause for concern, importantly these events were not associated with serious infections. This observation was also made in the larger, double-blind, placebo-controlled PALOMA-3 study [5]. The current US Prescribing Information for Palbociclib (Ibrance®) [18] recommends checking the absolute neutrophil count on day 1 and day 14 of the first two cycles of therapy. This monitoring schedule was chosen considering that the onset of neutropenia occurred early during treatment, with median time to any grade, Grade ≥3, and Grade 4 neutropenia being 20 days, 28 days, and 16 days, respectively. The study data demonstrate that there is no cumulative toxicity with regard to neutropenia; instead, its incidence decreases over time.

For a newly diagnosed patient with ER+/HER2– advanced breast cancer, the treating physician is faced with the decision of treating with endocrine therapy or chemotherapy. This decision is affected by factors such as menopausal status, ECOG performance status, sites of disease, and type and length of time from prior adjuvant therapy. Based on the data from PALOMA-1/TRIO-18, palbociclib received accelerated approval from the US FDA for use in these patients. The role of CDK 4/6 inhibition in ER+/HER2– breast cancer has now been further validated in the larger, PALOMA-3 study. The findings presented here confirm the consistent benefit of palbociclib and letrozole in several relevant clinical subgroups, including patients of older age, patients with both lobular and ductal carcinoma, and patients with various disease sites and prior adjuvant therapy. The PALOMA-1/TRIO-18 study was the first study to demonstrate a significant improvement in PFS with a novel agent in the first-line treatment of advanced ER+/HER2– breast cancer. These findings now await confirmation in the larger, phase III, double-blind, placebo-controlled PALOMA-2/TRIO-22 study which has completed enrollment and is awaiting analyses based on accruing events (NCT01740427).

## Conclusion

We performed an expanded analysis of the efficacy (PFS and CBR) as well as the safety of palbociclib plus letrozole in clinically relevant subgroups from the PALOMA-1/TRIO-18 trial. The degree of benefit in both median PFS and the CBR rate observed as a result of the addition of palbociclib to letrozole was consistent in nearly all subgroups analyzed and was consistent with that seen in the overall study population. The safety profile of the combination treatment in all subgroups was comparable to that in the overall safety population of the study. The frequency of Grade 3–4 neutropenia in the overall safety population of the study decreased over time. Neutropenia was not associated with serious infections or infectious complications.

## Additional file

Additional file 1: Table S1. Palbociclib dose modification scheme used in the PALOMA-1/TRIO-18 trial for managing neutropenia. **Table S2.** Baseline characteristics of the subgroup with visceral metastases (intention-to-treat population). **Table S3.** Most common all-cause adverse events that occurred in at least 10 % of patients <65 years of age in the palbociclib + letrozole arm of the safety population. **Table S4.** Most common all-cause adverse events that occurred in at least 10 % of patients ≥65 years of age in the palbociclib + letrozole arm of the safety population. **Table S5.** Most common all-cause adverse events that occurred in at least 10 % of patients with ductal carcinoma in the palbociclib + letrozole arm of the safety population. **Table S6.** Most common all-cause adverse events that occurred in at least 10 % of patients with lobular carcinoma in the palbociclib + letrozole arm of the safety population. **Table S7.** Most common all-cause adverse events that occurred in at least 10 % of patients with no prior neo-adjuvant/adjuvant systemic treatment in the palbociclib + letrozole arm of the safety population. **Table S8.** Most common all-cause adverse events that occurred in at least 10 % of patients with prior neo-adjuvant/adjuvant systemic treatment in the palbociclib + letrozole arm of the safety population. **Table S9.** Most common all-cause adverse events that occurred in at least 10 % of patients with bone-only metastases at baseline in the palbociclib + letrozole arm of the safety population. **Table S10.** Most common all-cause adverse events that occurred in at least 10 % of patients with visceral metastases in the palbociclib + letrozole arm of the safety population. **Table S11.** Most common all-cause adverse events that occurred in at least 10 % of patients with other metastases (bone with other non-visceral sites or other disease sites alone) in the palbociclib + letrozole arm of the safety population. (DOCX 74 kb)

## Abbrievations

AE: adverse event
CBR: clinical benefit response
CDK: cyclin-dependent kinase
CI: confidence interval
ECOG: Eastern Cooperative Oncology Group
ER: estrogen receptor
FISH: fluorescent in-situ hybridization
HER2: human epidermal growth factor receptor 2
HR: hazard ratio
HR+: hormone receptor-positive
ITT: intention-to-treat
PFS: progression-free survival
RECIST: Response Evaluation Criteria in Solid Tumors
RMSE: root mean square error
VIF: variance inflation factor
