# Supplementary material for: Efficacy and safety of palbociclib in combination with letrozole as first-line treatment of ER-positive, HER2-negative, advanced breast cancer: expanded analyses of subgroups from the randomized pivotal trial PALOMA-1/TRIO-18
Source: Breast Cancer Res. 2016 Jun 28;18:67. doi: 10.1186/s13058-016-0721-5 (PMC4924326; doi:10.1186/s13058-016-0721-5)
Supplement: Additional file 1: Table S1. — Palbociclib dose modification scheme used in the PALOMA-1/TRIO-18 trial for managing neutropenia. Table S2. Baseline characteristics of the subgroup with visceral metastases (intention-to-treat population). Table S3. Most common all-cause adverse events that occurred in at least 10 % of patients <65 years of age in the palbociclib + letrozole arm of the safety population. Table S4. Most common all-cause adverse events that occurred in at least 10 % of patients ≥65 years of age in the palbociclib + letrozole arm of the safety population. Table S5. Most common all-cause adverse events that occurred in at least 10 % of patients with ductal carcinoma in the palbociclib + letrozole arm of the safety population. Table S6. Most common all-cause adverse events that occurred in at least 10 % of patients with lobular carcinoma in the palbociclib + letrozole arm of the safety population. Table S7. Most common all-cause adverse events that occurred in at least 10 % of patients with no prior neo-adjuvant/adjuvant systemic treatment in the palbociclib + letrozole arm of the safety population. Table S8. Most common all-cause adverse events that occurred in at least 10 % of patients with prior neo-adjuvant/adjuvant systemic treatment in the palbociclib + letrozole arm of the safety population. Table S9. Most common all-cause adverse events that occurred in at least 10 % of patients with bone-only metastases at baseline in the palbociclib + letrozole arm of the safety population. Table S10. Most common all-cause adverse events that occurred in at least 10 % of patients with visceral metastases in the palbociclib + letrozole arm of the safety population. Table S11. Most common all-cause adverse events that occurred in at least 10 % of patients with other metastases (bone with other non-visceral sites or other disease sites alone) in the palbociclib + letrozole arm of the safety population. (DOCX 74 kb) [file 13058_2016_721_MOESM1_ESM.docx]

**Additional file 1**

Table S1: Palbociclib dose modification scheme used in the PALOMA-1/TRIO-18 trial for managing neutropenia

| **Worst toxicity during previous cycle** | **Dose modification** |
| --- | --- |
| Grade 4 neutropenia | Decrease 1 dose level |
| Grade 3 neutropenia associated with a documented infection or fever ≥38.5°C | Decrease 1 dose level |
| Persisting treatment-related toxicities  (ANC <1,000/mm^3^) resulting in delay by ≥1 week in receiving the next scheduled dose of either study treatment | If recovery occurs within a maximum of 2 weeks, continue and decrease 1 dose level.  If recovery does not occur within a maximum of 2 weeks, discontinue treatment. |
| AEs possibly related to study treatment resulting in inability to deliver ≥80% of the planned palbociclib or letrozole doses during cycle 2 | Decrease 1 dose level |

AE = adverse event; ANC = absolute neutrophil count.

Table S2: Baseline characteristics of the subgroup with visceral metastases (intention-to-treat population)

| **Visceral Metastases^a^** | **Involving Liver (n=42)** | | **Involving Lung (n=61)** | |
| --- | --- | --- | --- | --- |
|  | **P+ L (n=19)** | **L (n=23)** | **P+L (n=31)** | **L (n=30)** |
| Median Age (range), years | 61.0 (41-83) | 63.0 (50-84) | 65.0 (43-83) | 66.0 (38-78) |
| ECOG Performance Status:  0  1 | 11 (57.9%)  8 (42.1%) | 13 (56.5%)  10 (43.5%) | 13 (41.9%)  18 (58.1%) | 14 (46.7%)  16 (53.3%) |
| Disease Stage:  IIIB  IV | 0  19 (100.0%) | 0  23 (100.0%) | 0  31 (100.0%) | 0  30 (100.0%) |
| Prior Systemic Treatment:  None  Yes | 14 (73.7%)  5 (26.3%) | 8 (34.8%)  15 (65.2%) | 18 (58.1%)  13 (41.9%) | 11 (36.7%)  19 (63.3%) |

^a^based on case report form data; P+L: Palbociclib + Letrozole; L: Letrozole alone; Data are n (%) unless otherwise indicated; ECOG: Eastern Cooperative Oncology Group.

Table S3: Most common all-cause adverse events that occurred in at least 10% of patients <65 years of age in the palbociclib + letrozole arm of the safety population

|  | **Palbociclib + Letrozole**  **n = 46** | | | | | **Letrozole**  **n = 40** | | | | |
| --- | --- | --- | --- | --- | --- | --- | --- | --- | --- | --- |
| **Preferred Term** | **All Grades**  **n (%)** | **Grade 1**  **n (%)** | **Grade 2**  **n (%)** | **Grade 3**  **n (%)** | **Grade 4**  **n (%)** | **All Grades**  **n (%)** | **Grade 1**  **n (%)** | **Grade 2**  **n (%)** | **Grade 3**  **n (%)** | **Grade 4**  **n (%)** |
| Any AEs | 46 (100.0) | 0 (0.0) | 9 (19.6) | 30 (65.2) | 6 (13.0) | 33 (82.5) | 8 (20.0) | 21 (52.5) | 4 (10.0) | 0 (0.0) |
| Neutropenia | 32 (69.6) | 2 (4.3) | 6 (13.0) | 22 (47.8) | 2 (4.3) | 3 (7.5) | 1 (2.5) | 2 (5.0) | 0 (0.0) | 0 (0.0) |
| Fatigue | 17 (37.0) | 7 (15.2) | 10 (21.7) | 0 (0.0) | 0 (0.0) | 10 (25.0) | 6 (15.0) | 3 (7.5) | 1 (2.5) | 0 (0.0) |
| Leukopenia | 16 (34.8) | 3 (6.5) | 8 (17.4) | 5 (10.9) | 0 (0.0) | 2 (5.0) | 0 (0.0) | 2 (5.0) | 0 (0.0) | 0 (0.0) |
| Anemia | 13 (28.3) | 1 (2.2) | 8 (17.4) | 3 (6.5) | 1 (2.2) | 3 (7.5) | 1 (2.5) | 2 (5.0) | 0 (0.0) | 0 (0.0) |
| Hot flush | 12 (26.1) | 11 (23.9) | 1 (2.2) | 0 (0.0) | 0 (0.0) | 7 (17.5) | 5 (12.5) | 2 (5.0) | 0 (0.0) | 0 (0.0) |
| Arthralgia | 11 (23.9) | 5(10.9) | 5 (10.9) | 1 (2.2) | 0 (0.0) | 7 (17.5) | 5 (12.5) | 2 (5.0) | 0 (0.0) | 0 (0.0) |
| Nausea | 10 (21.7) | 9 (19.6) | 0 (0.0) | 1 (2.2) | 0 (0.0) | 6 (15.0) | 3 (7.5) | 3 (7.5) | 0 (0.0) | 0 (0.0) |
| Alopecia | 8 (17.4) | 8 (17.4) | 0 (0.0) | 0 (0.0) | 0 (0.0) | 1 (2.5) | 1 (2.5) | 0 (0.0) | 0 (0.0) | 0 (0.0) |
| Dyspnea | 8 (17.4) | 5 (10.9) | 1 (2.2) | 2 (4.3) | 0 (0.0) | 2 (5.0) | 2 (5.0) | 0 (0.0) | 0 (0.0) | 0 (0.0) |
| Nasopharyngitis | 8 (17.4) | 7 (15.2) | 1 (2.2) | 0 (0.0) | 0 (0.0) | 4 (10.0) | 2 (5.0) | 2 (5.0) | 0 (0.0) | 0 (0.0) |
| Decreased appetite | 7 (15.2) | 7 (15.2) | 0 (0.0) | 0 (0.0) | 0 (0.0) | 3 (7.5) | 3 (7.5) | 0 (0.0) | 0 (0.0) | 0 (0.0) |
| Diarrhea | 7 (15.2) | 3 (6.5) | 4 (8.7) | 0 (0.0) | 0 (0.0) | 3 (7.5) | 1 (2.5) | 2 (5.0) | 0 (0.0) | 0 (0.0) |
| Asthenia | 6 (13.0) | 2 (4.3) | 2 (4.3) | 2 (4.3) | 0 (0.0) | 2 (5.0) | 2 (5.0) | 0 (0.0) | 0 (0.0) | 0 (0.0) |
| Headache | 6 (13.0) | 4 (8.7) | 2 (4.3) | 0 (0.0) | 0 (0.0) | 7 (17.5) | 4 (10.0) | 3 (7.5) | 0 (0.0) | 0 (0.0) |
| Influenza | 6 (13.0) | 4 (8.7) | 2 (4.3) | 0 (0.0) | 0 (0.0) | 1 (2.5) | 0 (0.0) | 1 (2.5) | 0 (0.0) | 0 (0.0) |
| Insomnia | 6 (13.0) | 5 (10.9) | 1 (2.2) | 0 (0.0) | 0 (0.0) | 3 (7.5) | 2 (5.0) | 1 (2.5) | 0 (0.0) | 0 (0.0) |
| Stomatitis | 6 (13.0) | 3 (6.5) | 3 (6.5) | 0 (0.0) | 0 (0.0) | 1 (2.5) | 1 (2.5) | 0 (0.0) | 0 (0.0) | 0 (0.0) |
| Thrombocytopenia | 6 (13.0) | 3 (6.5) | 2 (4.3) | 1 (2.2) | 0 (0.0) | 0 (0.0) | 0 (0.0) | 0 (0.0) | 0 (0.0) | 0 (0.0) |
| Back pain | 5 (10.9) | 2 (4.3) | 3 (6.5) | 0 (0.0) | 0 (0.0) | 9 (22.5) | 5 (12.5) | 4 (10.0) | 0 (0.0) | 0 (0.0) |
| Bone pain | 5 (10.9) | 2 (4.3) | 3 (6.5) | 0 (0.0) | 0 (0.0) | 2 (5.0) | 1 (2.5) | 1 (2.5) | 0 (0.0) | 0 (0.0) |
| Cough | 5 (10.9) | 3 (6.5) | 2 (4.3) | 0 (0.0) | 0 (0.0) | 3 (7.5) | 3 (7.5) | 0 (0.0) | 0 (0.0) | 0 (0.0) |
| Upper respiratory tract infection | 5 (10.9) | 4 (8.7) | 1 (2.2) | 0 (0.0) | 0 (0.0) | 1 (2.5) | 0 (0.0) | 1 (2.5) | 0 (0.0) | 0 (0.0) |

Table S4: Most common all-cause adverse events that occurred in at least 10% of patients ≥65 years of age in the palbociclib + letrozole arm of the safety population

|  | **Palbociclib + Letrozole**  **n = 37** | | | | | **Letrozole**  **n = 37** | | | | |
| --- | --- | --- | --- | --- | --- | --- | --- | --- | --- | --- |
| **Preferred Term** | **All Grades**  **n (%)** | **Grade 1**  **n (%)** | **Grade 2**  **n (%)** | **Grade 3**  **n (%)** | **Grade 4**  **n (%)** | **All Grades**  **n (%)** | **Grade 1**  **n (%)** | **Grade 2**  **n (%)** | **Grade 3**  **n (%)** | **Grade 4**  **n (%)** |
| Any AEs | 37 (100.0) | 1 (2.7) | 9 (24.3) | 19 (51.4) | 8 (21.6) | 32 (86.5) | 9 (24.3) | 11 (29.7) | 12 (32.4) | 0 (0.0) |
| Neutropenia | 30 (81.1) | 0 (0.0) | 9 (24.3) | 18 (48.6) | 3 (8.1) | 1 (2.7) | 0 (0.0) | 0 (0.0) | 1 (2.7) | 0 (0.0) |
| Leukopenia | 20 (54.1) | 2 (5.4) | 7 (18.9) | 11 (29.7) | 0 (0.0) | 0 (0.0) | 0 (0.0) | 0 (0.0) | 0 (0.0) | 0 (0.0) |
| Fatigue | 17 (45.9) | 6 (16.2) | 7 (18.9) | 2 (5.4) | 2 (5.4) | 8 (21.6) | 5 (13.5) | 3 (8.1) | 0 (0.0) | 0 (0.0) |
| Anemia | 16 (43.2) | 3 (8.1) | 12 (32.4) | 1 (2.7) | 0 (0.0) | 2 (5.4) | 0 (0.0) | 1 (2.7) | 1 (2.7) | 0 (0.0) |
| Nausea | 11 (29.7) | 4 (10.8) | 6 (16.2) | 1 (2.7) | 0 (0.0) | 4 (10.8) | 2 (5.4) | 1 (2.7) | 1 (2.7) | 0 (0.0) |
| Alopecia | 10 (27.0) | 9 (24.3) | 1 (2.7) | 0 (0.0) | 0 (0.0) | 1 (2.7) | 1 (2.7) | 0 (0.0) | 0 (0.0) | 0 (0.0) |
| Diarrhea | 10 (27.0) | 5 (13.5) | 2 (5.4) | 3 (8.1) | 0 (0.0) | 5 (13.5) | 4 (10.8) | 1 (2.7) | 0 (0.0) | 0 (0.0) |
| Vomiting | 10 (27.0) | 6 (16.2) | 4 (10.8) | 0 (0.0) | 0 (0.0) | 3 (8.1) | 2 (5.4) | 0 (0.0) | 1 (2.7) | 0 (0.0) |
| Arthralgia | 8 (21.6) | 4 (10.8) | 4 (10.8) | 0 (0.0) | 0 (0.0) | 5 (13.5) | 0 (0.0) | 3 (8.1) | 2 (5.4) | 0 (0.0) |
| Thrombocytopenia | 8 (21.6) | 4 (10.8) | 3 (8.1) | 1 (2.7) | 0 (0.0) | 1 (2.7) | 1 (2.7) | 0 (0.0) | 0 (0.0) | 0 (0.0) |
| Back pain | 7 (18.9) | 5 (13.5) | 1 (2.7) | 0 (0.0) | 1 (2.7) | 3 (8.1) | 2 (5.4) | 0 (0.0) | 1 (2.7) | 0 (0.0) |
| Epistaxis | 7 (18.9) | 7 (18.9) | 0 (0.0) | 0 (0.0) | 0 (0.0) | 1 (2.7) | 1 (2.7) | 0 (0.0) | 0 (0.0) | 0 (0.0) |
| Pyrexia | 7 (18.9) | 7 (18.9) | 0 (0.0) | 0 (0.0) | 0 (0.0) | 1 (2.7) | 1 (2.7) | 0 (0.0) | 0 (0.0) | 0 (0.0) |
| Constipation | 6 (16.2) | 4 (10.8) | 2 (5.4) | 0 (0.0) | 0 (0.0) | 3 (8.1) | 1 (2.7) | 2 (5.4) | 0 (0.0) | 0 (0.0) |
| Decreased appetite | 6 (16.2) | 4 (10.8) | 1 (2.7) | 1 (2.7) | 0 (0.0) | 2 (5.4) | 1 (2.7) | 1 (2.7) | 0 (0.0) | 0 (0.0) |
| Headache | 6 (16.2) | 4 (10.8) | 2 (5.4) | 0 (0.0) | 0 (0.0) | 1 (2.7) | 0 (0.0) | 1 (2.7) | 0 (0.0) | 0 (0.0) |
| Asthenia | 5 (13.5) | 4 (10.8) | 1 (2.7) | 0 (0.0) | 0 (0.0) | 1 (2.7) | 1 (2.7) | 0 (0.0) | 0 (0.0) | 0 (0.0) |
| Bone pain | 5 (13.5) | 1 (2.7) | 2 (5.4) | 1 (2.7) | 1 (2.7) | 1 (2.7) | 1 (2.7) | 0 (0.0) | 0 (0.0) | 0 (0.0) |
| Cough | 5 (13.5) | 3 (8.1) | 2 (5.4) | 0 (0.0) | 0 (0.0) | 5 (13.5) | 4 (10.8) | 1 (2.7) | 0 (0.0) | 0 (0.0) |
| Dizziness | 5 (13.5) | 4 (10.8) | 1 (2.7) | 0 (0.0) | 0 (0.0) | 1 (2.7) | 1 (2.7) | 0 (0.0) | 0 (0.0) | 0 (0.0) |
| Dyspnea | 5 (13.5) | 2 (5.4) | 3 (8.1) | 0 (0.0) | 0 (0.0) | 4 (10.8) | 1 (2.7) | 2 (5.4) | 1 (2.7) | 0 (0.0) |
| Hot flush | 5 (13.5) | 5 (13.5) | 0 (0.0) | 0 (0.0) | 0 (0.0) | 2 (5.4) | 2 (5.4) | 0 (0.0) | 0 (0.0) | 0 (0.0) |
| Musculoskeletal pain | 5 (13.5) | 4 (10.8) | 1 (2.7) | 0 (0.0) | 0 (0.0) | 2 (5.4) | 1 (2.7) | 1 (2.7) | 0 (0.0) | 0 (0.0) |
| Nasopharyngitis | 5 (13.5) | 3 (8.1) | 2 (5.4) | 0 (0.0) | 0 (0.0) | 4 (10.8) | 3 (8.1) | 1 (2.7) | 0 (0.0) | 0 (0.0) |
| Oropharyngeal pain | 5 (13.5) | 4 (10.8) | 1 (2.7) | 0 (0.0) | 0 (0.0) | 0 (0.0) | 0 (0.0) | 0 (0.0) | 0 (0.0) | 0 (0.0) |
| Rash | 5 (13.5) | 4 (10.8) | 1 (2.7) | 0 (0.0) | 0 (0.0) | 1 (2.7) | 1 (2.7) | 0 (0.0) | 0 (0.0) | 0 (0.0) |
| Urinary tract infection | 5 (13.5) | 0 (0.0) | 5 (13.5) | 0 (0.0) | 0 (0.0) | 3 (8.1) | 1 (2.7) | 2 (5.4) | 0 (0.0) | 0 (0.0) |
| Dysgeusia | 4 (10.8) | 4 (10.8) | 0 (0.0) | 0 (0.0) | 0 (0.0) | 0 (0.0) | 0 (0.0) | 0 (0.0) | 0 (0.0) | 0 (0.0) |
| Fall | 4 (10.8) | 3 (8.1) | 1 (2.7) | 0 (0.0) | 0 (0.0) | 2 (5.4) | 1 (2.7) | 1 (2.7) | 0 (0.0) | 0 (0.0) |
| Hypertension | 4 (10.8) | 0 (0.0) | 4 (10.8) | 0 (0.0) | 0 (0.0) | 0 (0.0) | 0 (0.0) | 0 (0.0) | 0 (0.0) | 0 (0.0) |
| Neuropathy peripheral | 4 (10.8) | 3 (8.1) | 1 (2.7) | 0 (0.0) | 0 (0.0) | 1 (2.7) | 0 (0.0) | 1 (2.7) | 0 (0.0) | 0 (0.0) |
| Pain in extremity | 4 (10.8) | 1 (2.7) | 3 (8.1) | 0 (0.0) | 0 (0.0) | 3 (8.1) | 3 (8.1) | 0 (0.0) | 0 (0.0) | 0 (0.0) |
| Stomatitis | 4 (10.8) | 1 (2.7) | 3 (8.1) | 0 (0.0) | 0 (0.0) | 1 (2.7) | 1 (2.7) | 0 (0.0) | 0 (0.0) | 0 (0.0) |
| Upper respiratory tract infection | 4 (10.8) | 1 (2.7) | 2 (5.4) | 1 (2.7) | 0 (0.0) | 1 (2.7) | 0 (0.0) | 1 (2.7) | 0 (0.0) | 0 (0.0) |

Table S5: Most common all-cause adverse events that occurred in at least 10% of patients with ductal carcinoma in the palbociclib + letrozole arm of the safety population

|  | **Palbociclib + Letrozole**  **n = 62** | | | | | **Letrozole**  **n = 51** | | | | |
| --- | --- | --- | --- | --- | --- | --- | --- | --- | --- | --- |
| **Preferred Term** | **All Grades**  **n (%)** | **Grade 1**  **n (%)** | **Grade 2**  **n (%)** | **Grade 3**  **n (%)** | **Grade 4**  **n (%)** | **All Grades**  **n (%)** | **Grade 1**  **n (%)** | **Grade 2**  **n (%)** | **Grade 3**  **n (%)** | **Grade 4**  **n (%)** |
| Any AEs | 62 (100.0) | 0 (0.0) | 15 (24.2) | 35 (56.5) | 12 (19.4) | 44 (86.3) | 13 (25.5) | 18 (35.3) | 13 (25.5) | 0 (0.0) |
| Neutropenia | 48 (77.4) | 2 (3.2) | 11 (17.7) | 31 (50.0) | 4 (6.5) | 3 (5.9) | 1 (2.0) | 1 (2.0) | 1 (2.0) | 0 (0.0) |
| Fatigue | 28 (45.2) | 10 (16.1) | 14 (22.6) | 2 (3.2) | 2 (3.2) | 13 (25.5) | 7 (13.7) | 5 (9.8) | 1 (2.0) | 0 (0.0) |
| Leukopenia | 26 (41.9) | 3 (4.8) | 8 (12.9) | 15 (24.2) | 0 (0.0) | 1 (2.0) | 0 (0.0) | 1 (2.0) | 0 (0.0) | 0 (0.0) |
| Anemia | 19 (30.6) | 3 (4.8) | 14 (22.6) | 1 (1.6) | 1 (1.6) | 4 (7.8) | 1 (2.0) | 2 (3.9) | 1 (2.0) | 0 (0.0) |
| Nausea | 16 (25.8) | 12 (19.4) | 3 (4.8) | 1 (1.6) | 0 (0.0) | 6 (11.8) | 3 (5.9) | 2 (3.9) | 1 (2.0) | 0 (0.0) |
| Alopecia | 15 (24.2) | 14 (22.6) | 1 (1.6) | 0 (0.0) | 0 (0.0) | 2 (3.9) | 2 (3.9) | 0 (0.0) | 0 (0.0) | 0 (0.0) |
| Arthralgia | 15 (24.2) | 7 (11.3) | 8 (12.9) | 0 (0.0) | 0 (0.0) | 10 (19.6) | 4 (7.8) | 4 (7.8) | 2 (3.9) | 0 (0.0) |
| Hot flush | 15 (24.2)) | 14 (22.6) | 1 (1.6) | 0 (0.0) | 0 (0.0) | 8 (15.7) | 6 (11.8) | 2 (3.9) | 0 (0.0) | 0 (0.0) |
| Diarrhea | 14 (22.6) | 5 (8.1) | 6 (9.7) | 3 (4.8) | 0 (0.0) | 7 (13.7) | 5 (9.8) | 2 (3.9) | 0 (0.0) | 0 (0.0) |
| Decreased appetite | 12 (19.4) | 10 (16.1) | 1 (1.6) | 1 (1.6) | 0 (0.0) | 3 (5.9) | 3 (5.9) | 0 (0.0) | 0 (0.0) | 0 (0.0) |
| Thrombocytopenia | 12 (19.4) | 6 (9.7) | 4 (6.5) | 2 (3.2) | 0 (0.0) | 1 (2.0) | 1 (2.0) | 0 (0.0) | 0 (0.0) | 0 (0.0) |
| Bone pain | 10 (16.1) | 3 (4.8) | 5 (8.1) | 1 (1.6) | 1 (1.6) | 1 (2.0) | 1 (2.0) | 0 (0.0) | 0 (0.0) | 0 (0.0) |
| Dyspnea | 10 (16.1 | 5 (8.1) | 3 (4.8) | 2 (3.2) | 0 (0.0) | 5 (9.8) | 3 (5.9) | 1 (2.0) | 1 (2.0) | 0 (0.0) |
| Nasopharyngitis | 10 (16.1 | 9 (14.5) | 1 (1.6) | 0 (0.0) | 0 (0.0) | 6 (11.8) | 4 (7.8) | 2 (3.9) | 0 (0.0) | 0 (0.0) |
| Back pain | 9 (14.5) | 5 (8.1) | 3 (4.8) | 0 (0.0) | 1 (1.6) | 8 (15.7) | 6 (11.8) | 2 (3.9) | 0 (0.0) | 0 (0.0) |
| Cough | 9 (14.5) | 5 (8.1) | 4 (6.5) | 0 (0.0) | 0 (0.0) | 6 (11.8) | 5 (9.8) | 1 (2.0) | 0 (0.0) | 0 (0.0) |
| Headache | 9 (14.5) | 6 (9.7) | 3 (4.8) | 0 (0.0) | 0 (0.0) | 6 (11.8) | 2 (3.9) | 4 (7.8) | 0 (0.0) | 0 (0.0) |
| Constipation | 8 (12.9) | 6 (9.7) | 2 (3.2) | 0 (0.0) | 0 (0.0) | 5 (9.8) | 2 (3.9) | 3 (5.9) | 0 (0.0) | 0 (0.0) |
| Dizziness | 8 (12.9) | 7 (11.3) | 1 (1.6) | 0 (0.0) | 0 (0.0) | 2 (3.9) | 2 (3.9) | 0 (0.0) | 0 (0.0) | 0 (0.0) |
| Musculoskeletal pain | 8 (12.9) | 5 (8.1) | 2 (3.2) | 1 (1.6) | 0 (0.0) | 2 (3.9) | 2 (3.9) | 0 (0.0) | 0 (0.0) | 0 (0.0) |
| Vomiting | 8 (12.9) | 6 (9.7) | 2 (3.2) | 0 (0.0) | 0 (0.0) | 2 (3.9) | 1 (2.0) | 0 (0.0) | 1 (2.0) | 0 (0.0) |
| Epistaxis | 7 (11.3) | 7 (11.3) | 0 (0.0) | 0 (0.0) | 0 (0.0) | 1 (2.0) | 1 (2.0) | 0 (0.0) | 0 (0.0) | 0 (0.0) |
| Influenza | 7 (11.3) | 3 (4.8) | 4 (6.5) | 0 (0.0) | 0 (0.0) | 1 (2.0) | 0 (0.0) | 1 (2.0) | 0 (0.0) | 0 (0.0) |
| Pain in extremity | 7 (11.3) | 5 (8.1) | 2 (3.2) | 0 (0.0) | 0 (0.0) | 4 (7.8) | 3 (5.9) | 1 (2.0) | 0 (0.0) | 0 (0.0) |
| Stomatitis | 7 (11.3) | 3 (4.8) | 4 (6.5) | 0 (0.0) | 0 (0.0) | 2 (3.9) | 2 (3.9) | 0 (0.0) | 0 (0.0) | 0 (0.0) |
| Upper respiratory tract infection | 7 (11.3) | 4 (6.5) | 3 (4.8) | 0 (0.0) | 0 (0.0) | 1 (2.0) | 0 (0.0) | 1 (2.0) | 0 (0.0) | 0 (0.0) |

Table S6: Most common all-cause adverse events that occurred in at least 10% of patients with lobular carcinoma in the palbociclib + letrozole arm of the safety population

|  | **Palbociclib + Letrozole**  **n = 18** | | | | | **Letrozole**  **n = 18** | | | | |
| --- | --- | --- | --- | --- | --- | --- | --- | --- | --- | --- |
| **Preferred Term** | **All Grades**  **n (%)** | **Grade 1**  **n (%)** | **Grade 2**  **n (%)** | **Grade 3**  **n (%)** | **Grade 4**  **n (%)** | **All Grades**  **n (%)** | **Grade 1**  **n (%)** | **Grade 2**  **n (%)** | **Grade 3**  **n (%)** | **Grade 4**  **n (%)** |
| Any AEs | 18 (100.0) | 1 (5.6) | 3 (16.7) | 12 (66.7) | 1 (5.6) | 13 (72.2) | 3 (16.7) | 8 (44.4) | 2 (11.1) | 0 (0.0) |
| Neutropenia | 12 (66.7) | 0 (0.0) | 4 (22.2) | 7 (38.9) | 1 (5.6) | 1 (5.6) | 0 (0.0) | 1 (5.6) | 0 (0.0) | 0 (0.0) |
| Anemia | 8 (44.4) | 1 (5.6) | 5 (27.8) | 2 (11.1) | 0 (0.0) | 1 (5.6) | 0 (0.0) | 1 (5.6) | 0 (0.0) | 0 (0.0) |
| Leukopenia | 8 (44.4) | 2 (11.1) | 5 (27.8) | 1 (5.6) | 0 (0.0) | 1 (5.6) | 0 (0.0) | 1 (5.6) | 0 (0.0) | 0 (0.0) |
| Asthenia | 5 (27.8) | 2 (11.1) | 1 (5.6) | 2 (11.1) | 0 (0.0) | 0 (0.0) | 0 (0.0) | 0 (0.0) | 0 (0.0) | 0 (0.0) |
| Blood alkaline phosphatase increased | 4 (22.2) | 1 (5.6) | 3 (16.7) | 0 (0.0) | 0 (0.0) | 1 (5.6) | 1 (5.6) | 0 (0.0) | 0 (0.0) | 0 (0.0) |
| Fatigue | 4 (22.2) | 2 (11.1) | 2 (11.1) | 0 (0.0) | 0 (0.0) | 3 (16.7) | 3 (16.7) | 0 (0.0) | 0 (0.0) | 0 (0.0) |
| Nausea | 4 (22.2) | 1 (5.6) | 2 (11.1) | 1 (5.6) | 0 (0.0) | 3 (16.7) | 2 (11.1) | 1 (5.6) | 0 (0.0) | 0 (0.0) |
| Alopecia | 3 (16.7) | 3 (16.7) | 0 (0.0) | 0 (0.0) | 0 (0.0) | 0 (0.0) | 0 (0.0) | 0 (0.0) | 0 (0.0) | 0 (0.0) |
| Arthralgia | 3 (16.7) | 2 (11.1) | 0 (0.0) | 1 (5.6) | 0 (0.0) | 0 (0.0) | 0 (0.0) | 0 (0.0) | 0 (0.0) | 0 (0.0) |
| Aspartate aminotransferase increased | 3 (16.7) | 1 (5.6) | 1 (5.6) | 1 (5.6) | 0 (0.0) | 0 (0.0) | 0 (0.0) | 0 (0.0) | 0 (0.0) | 0 (0.0) |
| Neuropathy peripheral | 3 (16.7) | 2 (11.1) | 1 (5.6) | 0 (0.0) | 0 (0.0) | 0 (0.0) | 0 (0.0) | 0 (0.0) | 0 (0.0) | 0 (0.0) |
| Edema peripheral | 3 (16.7) | 1 (5.6) | 2 (11.1) | 0 (0.0) | 0 (0.0) | 1 (5.6) | 1 (5.6) | 0 (0.0) | 0 (0.0) | 0 (0.0) |
| Vomiting | 3 (16.7) | 1 (5.6) | 2 (11.1) | 0 (0.0) | 0 (0.0) | 1 (5.6) | 1 (5.6) | 0 (0.0) | 0 (0.0) | 0 (0.0) |
| Abdominal pain | 2 (11.1) | 1 (5.6) | 1 (5.6) | 0 (0.0) | 0 (0.0) | 1 (5.6) | 1 (5.6) | 0 (0.0) | 0 (0.0) | 0 (0.0) |
| Alanine aminotransferase increased | 2 (11.1) | 1 (5.6) | 1 (5.6) | 0 (0.0) | 0 (0.0) | 0 (0.0) | 0 (0.0) | 0 (0.0) | 0 (0.0) | 0 (0.0) |
| Diarrhea | 2 (11.1) | 2 (11.1) | 0 (0.0) | 0 (0.0) | 0 (0.0) | 0 (0.0) | 0 (0.0) | 0 (0.0) | 0 (0.0) | 0 (0.0) |
| Dyspnea | 2 (11.1) | 2 (11.1) | 0 (0.0) | 0 (0.0) | 0 (0.0) | 0 (0.0) | 0 (0.0) | 0 (0.0) | 0 (0.0) | 0 (0.0) |
| Headache | 2 (11.1) | 1 (5.6) | 1 (5.6) | 0 (0.0) | 0 (0.0) | 0 (0.0) | 0 (0.0) | 0 (0.0) | 0 (0.0) | 0 (0.0) |
| Hot flush | 2 (11.1) | 2 (11.1) | 0 (0.0) | 0 (0.0) | 0 (0.0) | 0 (0.0) | 0 (0.0) | 0 (0.0) | 0 (0.0) | 0 (0.0) |
| Hyperuricemia | 2 (11.1) | 2 (11.1) | 0 (0.0) | 0 (0.0) | 0 (0.0) | 1 (5.6) | 1 (5.6) | 0 (0.0) | 0 (0.0) | 0 (0.0) |
| Influenza-like illness | 2 (11.1) | 2 (11.1) | 0 (0.0) | 0 (0.0) | 0 (0.0) | 0 (0.0) | 0 (0.0) | 0 (0.0) | 0 (0.0) | 0 (0.0) |
| Stomatitis | 2 (11.1) | 1 (5.6) | 1 (5.6) | 0 (0.0) | 0 (0.0) | 0 (0.0) | 0 (0.0) | 0 (0.0) | 0 (0.0) | 0 (0.0) |
| Thrombocytopenia | 2 (11.1) | 1 (5.6) | 1 (5.6) | 0 (0.0) | 0 (0.0) | 0 (0.0) | 0 (0.0) | 0 (0.0) | 0 (0.0) | 0 (0.0) |
| Urinary tract infection | 2 (11.1) | 0 (0.0) | 2 (11.1) | 0 (0.0) | 0 (0.0) | 0 (0.0) | 0 (0.0) | 0 (0.0) | 0 (0.0) | 0 (0.0) |
| Visual impairment | 2 (11.1) | 2 (11.1) | 0 (0.0) | 0 (0.0) | 0 (0.0) | 0 (0.0) | 0 (0.0) | 0 (0.0) | 0 (0.0) | 0 (0.0) |

Table S7: Most common all-cause adverse events that occurred in at least 10% of patients with no prior neo-adjuvant/adjuvant systemic treatment in the palbociclib + letrozole arm of the safety population

|  | **Palbociclib + Letrozole**  **n = 43** | | | | | **Letrozole**  **n = 37** | | | | |
| --- | --- | --- | --- | --- | --- | --- | --- | --- | --- | --- |
| **Preferred Term** | **All Grades**  **n (%)** | **Grade 1**  **n (%)** | **Grade 2**  **n (%)** | **Grade 3**  **n (%)** | **Grade 4**  **n (%)** | **All Grades**  **n (%)** | **Grade 1**  **n (%)** | **Grade 2**  **n (%)** | **Grade 3**  **n (%)** | **Grade 4**  **n (%)** |
| Any AEs | 43 (100.0) | 1 (2.3) | 12 (27.9) | 22 (51.2) | 7 (16.3) | 32 (86.5) | 9 (24.3) | 18 (48.6) | 5 (13.5) | 0 (0.0) |
| Neutropenia | 29 (67.4) | 1 (2.3) | 6 (14.0) | 19 (44.2) | 3 (7.0) | 3 (8.1) | 1 (2.7) | 2 (5.4) | 0 (0.0) | 0 (0.0) |
| Fatigue | 21 (48.8) | 9 (20.9) | 11 (25.6) | 0 (0.0) | 1 (2.3) | 7 (18.9) | 4 (10.8) | 2 (5.4) | 1 (2.7) | 0 (0.0) |
| Leukopenia | 21 (48.8) | 5 (11.6) | 7 (16.3) | 9 (20.9) | 0 (0.0) | 2 (5.4) | 0 (0.0) | 2 (5.4) | 0 (0.0) | 0 (0.0) |
| Anemia | 15 (34.9) | 2 (4.7) | 11 (25.6) | 2 (4.7) | 0 (0.0) | 2 (5.4) | 1 (2.7) | 1 (2.7) | 0 (0.0) | 0 (0.0) |
| Alopecia | 13 (30.2) | 12 (27.9) | 1 (2.3) | 0 (0.0) | 0 (0.0) | 2 (5.4) | 2 (5.4) | 0 (0.0) | 0 (0.0) | 0 (0.0) |
| Arthralgia | 10 (23.3) | 5 (11.6) | 5 (11.6) | 0 (0.0) | 0 (0.0) | 5 (13.5) | 2 (5.4) | 2 (5.4) | 1 (2.7) | 0 (0.0) |
| Hot flush | 10 (23.3) | 9 (20.9) | 1 (2.3) | 0 (0.0) | 0 (0.0) | 6 (16.2) | 4 (10.8) | 2 (5.4) | 0 (0.0) | 0 (0.0) |
| Diarrhea | 8 (18.6) | 3 (7.0) | 3 (7.0) | 2 (4.7) | 0 (0.0) | 2 (5.4) | 2 (5.4) | 0 (0.0) | 0 (0.0) | 0 (0.0) |
| Nausea | 8 (18.6) | 7 (16.3) | 1 (2.3) | 0 (0.0) | 0 (0.0) | 5 (13.5) | 1 (2.7) | 3 (8.1) | 1 (2.7) | 0 (0.0) |
| Decreased appetite | 7 (16.3) | 7 (16.3) | 0 (0.0) | 0 (0.0) | 0 (0.0) | 2 (5.4) | 2 (5.4) | 0 (0.0) | 0 (0.0) | 0 (0.0) |
| Dyspnea | 7 (16.3) | 5 (11.6) | 2 (4.7) | 0 (0.0) | 0 (0.0) | 2 (5.4) | 1 (2.7) | 1 (2.7) | 0 (0.0) | 0 (0.0) |
| Headache | 7 (16.3) | 5 (11.6) | 2 (4.7) | 0 (0.0) | 0 (0.0) | 3 (8.1) | 2 (5.4) | 1 (2.7) | 0 (0.0) | 0 (0.0) |
| Nasopharyngitis | 7 (16.3) | 5 (11.6) | 2 (4.7) | 0 (0.0) | 0 (0.0) | 4 (10.8) | 3 (8.1) | 1 (2.7) | 0 (0.0) | 0 (0.0) |
| Back pain | 6 (14.0) | 3 (7.0) | 3 (7.0) | 0 (0.0) | 0 (0.0) | 6 (16.2) | 2 (5.4) | 4 (10.8) | 0 (0.0) | 0 (0.0) |
| Bone pain | 6 (14.0) | 2 (4.7) | 3 (7.0) | 1 (2.3) | 0 (0.0) | 2 (5.4) | 1 (2.7) | 1 (2.7) | 0 (0.0) | 0 (0.0) |
| Influenza | 6 (14.0) | 3 (7.0) | 3 (7.0) | 0 (0.0) | 0 (0.0) | 0 (0.0) | 0 (0.0) | 0 (0.0) | 0 (0.0) | 0 (0.0) |
| Musculoskeletal pain | 6 (14.0) | 4 (9.3) | 1 (2.3) | 1 (2.3) | 0 (0.0) | 3 (8.1) | 2 (5.4) | 1 (2.7) | 0 (0.0) | 0 (0.0) |
| Stomatitis | 6 (14.0) | 3 (7.0) | 3 (7.0) | 0 (0.0) | 0 (0.0) | 1 (2.7) | 1 (2.7) | 0 (0.0) | 0 (0.0) | 0 (0.0) |
| Upper respiratory tract infection | 6 (14.0) | 4 (9.3) | 2 (4.7) | 0 (0.0) | 0 (0.0) | 1 (2.7) | 0 (0.0) | 1 (2.7) | 0 (0.0) | 0 (0.0) |
| Cough | 5 (11.6) | 3 (7.0) | 2 (4.7) | 0 (0.0) | 0 (0.0) | 1 (2.7) | 1 (2.7) | 0 (0.0) | 0 (0.0) | 0 (0.0) |
| Dizziness | 5 (11.6) | 4 (9.3) | 1 (2.3) | 0 (0.0) | 0 (0.0) | 1 (2.7) | 1 (2.7) | 0 (0.0) | 0 (0.0) | 0 (0.0) |
| Hypertension | 5 (11.6) | 0 (0.0) | 5 (11.6) | 0 (0.0) | 0 (0.0) | 2 (5.4) | 0 (0.0) | 2 (5.4) | 0 (0.0) | 0 (0.0) |
| Oropharyngeal pain | 5 (11.6) | 2 (4.7) | 3 (7.0) | 0 (0.0) | 0 (0.0) | 1 (2.7) | 1 (2.7) | 0 (0.0) | 0 (0.0) | 0 (0.0) |
| Pain in extremity | 5 (11.6) | 4 (9.3) | 1 (2.3) | 0 (0.0) | 0 (0.0) | 4 (10.8) | 3 (8.1) | 1 (2.7) | 0 (0.0) | 0 (0.0) |
| Thrombocytopenia | 5 (11.6) | 4 (9.3) | 1 (2.3) | 0 (0.0) | 0 (0.0) | 1 (2.7) | 1 (2.7) | 0 (0.0) | 0 (0.0) | 0 (0.0) |

Table S8: Most common all-cause adverse events that occurred in at least 10% of patients with prior neo-adjuvant/adjuvant systemic treatment in the palbociclib + letrozole arm of the safety population

|  | **Palbociclib + Letrozole**  **n = 40** | | | | | **Letrozole**  **n = 40** | | | | |
| --- | --- | --- | --- | --- | --- | --- | --- | --- | --- | --- |
| **Preferred Term** | **All Grades**  **n (%)** | **Grade 1**  **n (%)** | **Grade 2**  **n (%)** | **Grade 3**  **n (%)** | **Grade 4**  **n (%)** | **All Grades**  **n (%)** | **Grade 1**  **n (%)** | **Grade 2**  **n (%)** | **Grade 3**  **n (%)** | **Grade 4**  **n (%)** |
| Any AEs | 40 (100.0) | 0 (0.0) | 6 (15.0) | 27 (67.5) | 7 (17.5) | 33 (82.5) | 8 (20.0) | 14 (35.0) | 11 (27.5) | 0 (0.0) |
| Neutropenia | 33 (82.5) | 1 (2.5) | 9 (22.5) | 21 (52.5) | 2 (5.0) | 1 (2.5) | 0 (0.0) | 0 (0.0) | 1 (2.5) | 0 (0.0) |
| Leukopenia | 15 (37.5) | 0 (0.0) | 8 (20.0) | 7 (17.5) | 0 (0.0) | 0 (0.0) | 0 (0.0) | 0 (0.0) | 0 (0.0) | 0 (0.0) |
| Anemia | 14 (35.0) | 2 (5.0) | 9 (22.5) | 2 (5.0) | 1 (2.5) | 3 (7.5) | 0 (0.0) | 2 (5.0) | 1 (2.5) | 0 (0.0) |
| Fatigue | 13 (32.5) | 4 (10.0) | 6 (15.0) | 2 (5.0) | 1 (2.5) | 11 (27.5) | 7 (17.5) | 4 (10.0) | 0 (0.0) | 0 (0.0) |
| Nausea | 13 (32.5) | 6 (15.0) | 5 (12.5) | 2 (5.0) | 0 (0.0) | 5 (12.5) | 4 (10.0) | 1 (2.5) | 0 (0.0) | 0 (0.0) |
| Arthralgia | 9 (22.5) | 4 (10.0) | 4 (10.0) | 1 (2.5) | 0 (0.0) | 7 (17.5) | 3 (7.5) | 3 (7.5) | 1 (2.5) | 0 (0.0) |
| Diarrhea | 9 (22.5) | 5 (12.5) | 3 (7.5) | 1 (2.5) | 0 (0.0) | 6 (15.0) | 3 (7.5) | 3 (7.5) | 0 (0.0) | 0 (0.0) |
| Thrombocytopenia | 9 (22.5) | 3 (7.5) | 4 (10.0) | 2 (5.0) | 0 (0.0) | 0 (0.0) | 0 (0.0) | 0 (0.0) | 0 (0.0) | 0 (0.0) |
| Asthenia | 8 (20.0) | 4 (10.0) | 2 (5.0) | 2 (5.0) | 0 (0.0) | 0 (0.0) | 0 (0.0) | 0 (0.0) | 0 (0.0) | 0 (0.0) |
| Vomiting | 8 (20.0) | 4 (10.0) | 4 (10.0) | 0 (0.0) | 0 (0.0) | 2 (5.0) | 2 (5.0) | 0 (0.0) | 0 (0.0) | 0 (0.0) |
| Hot flush | 7 (17.5) | 7 (17.5) | 0 (0.0) | 0 (0.0) | 0 (0.0) | 3 (7.5) | 3 (7.5) | 0 (0.0) | 0 (0.0) | 0 (0.0) |
| Back pain | 6 (15.0) | 4 (10.0) | 1 (2.5) | 0 (0.0) | 1 (2.5) | 6 (15.0) | 5 (12.5) | 0 (0.0) | 1 (2.5) | 0 (0.0) |
| Constipation | 6 (15.0) | 4 (10.0) | 2 (5.0) | 0 (0.0) | 0 (0.0) | 2 (5.0) | 1 (2.5) | 1(2.5) | 0 (0.0) | 0 (0.0) |
| Decreased appetite | 6 (15.0) | 4 (10.0) | 1 (2.5) | 1 (2.5) | 0 (0.0) | 3 (7.5) | 2 (5.0) | 1 (2.5) | 0 (0.0) | 0 (0.0) |
| Dyspnea | 6 (15.0) | 2 (5.0) | 2 (5.0) | 2 (5.0) | 0 (0.0) | 4 (10.0) | 2 (5.0) | 1 (2.5) | 1 (2.5) | 0 (0.0) |
| Nasopharyngitis | 6 (15.0) | 5 (12.5) | 1 (2.5) | 0 (0.0) | 0 (0.0) | 4 (10.0) | 2 (5.0) | 2 (5.0) | 0 (0.0) | 0 (0.0) |
| Alopecia | 5 (12.5) | 5 (12.5) | 0 (0.0) | 0 (0.0) | 0 (0.0) | 0 (0.0) | 0 (0.0) | 0 (0.0) | 0 (0.0) | 0 (0.0) |
| Cough | 5 (12.5) | 3 (7.5) | 2 (5.0) | 0 (0.0) | 0 (0.0) | 7 (17.5) | 6 (15.0) | 1 (2.5) | 0 (0.0) | 0 (0.0) |
| Epistaxis | 5 (12.5) | 5 (12.5) | 0 (0.0) | 0 (0.0) | 0 (0.0) | 1 (2.5) | 1 (2.5) | 0 (0.0) | 0 (0.0) | 0 (0.0) |
| Headache | 5 (12.5) | 3 (7.5) | 2 (5.0) | 0 (0.0) | 0 (0.0) | 5 (12.5) | 2 (5.0) | 3 (7.5) | 0 (0.0) | 0 (0.0) |
| Mucosal inflammation | 5 (12.5) | 3 (7.5) | 2 (5.0) | 0 (0.0) | 0 (0.0) | 2 (5.0) | 1 (2.5) | 0 (0.0) | 1 (2.5) | 0 (0.0) |
| Neuropathy peripheral | 5 (12.5) | 3 (7.5) | 2 (5.0) | 0 (0.0) | 0 (0.0) | 1 (2.5) | 1 (2.5) | 0 (0.0) | 0 (0.0) | 0 (0.0) |
| Pyrexia | 5 (12.5) | 5 (12.5) | 0 (0.0) | 0 (0.0) | 0 (0.0) | 1 (2.5) | 1 (2.5) | 0 (0.0) | 0 (0.0) | 0 (0.0) |
| Urinary tract infection | 5 (12.5) | 0 (0.0) | 5 (12.5) | 0 (0.0) | 0 (0.0) | 3 (7.5) | 1 (2.5) | 2 (5.0) | 0 (0.0) | 0 (0.0) |
| Alanine aminotransferase increased | 4 (10.0) | 3 (7.5) | 1 (2.5) | 0 (0.0) | 0 (0.0) | 1 (2.5) | 0 (0.0) | 1 (2.5) | 0 (0.0) | 0 (0.0) |
| Bone pain | 4 (10.0) | 1 (2.5) | 2 (5.0) | 0 (0.0) | 1 (2.5) | 1 (2.5) | 1 (2.5) | 0 (0.0) | 0 (0.0) | 0 (0.0) |
| Hyperhidrosis | 4 (10.0) | 4 (10.0) | 0 (0.0) | 0 (0.0) | 0 (0.0) | 0 (0.0) | 0 (0.0) | 0 (0.0) | 0 (0.0) | 0 (0.0) |
| Stomatitis | 4 (10.0) | 1 (2.5) | 3 (7.5) | 0 (0.0) | 0 (0.0) | 1 (2.5) | 1 (2.5) | 0 (0.0) | 0 (0.0) | 0 (0.0) |

Table S9: Most common all-cause adverse events that occurred in at least 10% of patients with bone-only metastases at baseline in the palbociclib + letrozole arm of the safety population

|  | **Palbociclib + Letrozole**  **n = 17** | | | | | **Letrozole**  **n = 11** | | | | |
| --- | --- | --- | --- | --- | --- | --- | --- | --- | --- | --- |
| **Preferred Term** | **All Grades**  **n (%)** | **Grade 1**  **n (%)** | **Grade 2**  **n (%)** | **Grade 3**  **n (%)** | **Grade 4**  **n (%)** | **All Grades**  **n (%)** | **Grade 1**  **n (%)** | **Grade 2**  **n (%)** | **Grade 3**  **n (%)** | **Grade 4**  **n (%)** |
| Any AEs | 17 (100.0) | 0 (0.0) | 2 (11.8) | 12 (70.6) | 3 (17.6) | 10 (90.9) | 2 (18.2) | 6 (54.5) | 2 (18.2) | 0 (0.0) |
| Neutropenia | 14 (82.4) | 0 (0.0) | 2 (11.8) | 11 (64.7) | 1 (5.9) | 0 (0.0) | 0 (0.0) | 0 (0.0) | 0 (0.0) | 0 (0.0) |
| Fatigue | 10 (58.8) | 4 (23.5) | 5 (29.4) | 1 (5.9) | 0 (0.0) | 3 (27.3) | 2 (18.2) | 1 (9.1) | 0 (0.0) | 0 (0.0) |
| Leukopenia | 7 (41.2) | 0 (0.0) | 3 (17.6) | 4 (23.5) | 0 (0.0) | 0 (0.0) | 0 (0.0) | 0 (0.0) | 0 (0.0) | 0 (0.0) |
| Nausea | 7 (41.2) | 3 (17.6) | 4 (23.5) | 0 (0.0) | 0 (0.0) | 2 (18.2) | 0 (0.0) | 1 (9.1) | 1 (9.1) | 0 (0.0) |
| Anemia | 6 (35.3) | 0 (0.0) | 4 (23.5) | 1 (5.9) | 1 (5.9) | 1 (9.1) | 1 (9.1) | 0 (0.0) | 0 (0.0) | 0 (0.0) |
| Alopecia | 5 (29.4) | 5 (29.4) | 0 (0.0) | 0 (0.0) | 0 (0.0) | 1 (9.1) | 1 (9.1) | 0 (0.0) | 0 (0.0) | 0 (0.0) |
| Arthralgia | 5 (29.4) | 2 (11.8) | 2 (11.8) | 1 (5.9) | 0 (0.0) | 3 (27.3) | 0 (0.0) | 3 (27.3) | 0 (0.0) | 0 (0.0) |
| Diarrhea | 5 (29.4) | 3 (17.6) | 2 (11.8) | 0 (0.0) | 0 (0.0) | 1 (9.1) | 1 (9.1) | 0 (0.0) | 0 (0.0) | 0 (0.0) |
| Oropharyngeal pain | 5 (29.4) | 3 (17.6) | 2 (11.8) | 0 (0.0) | 0 (0.0) | 0 (0.0) | 0 (0.0) | 0 (0.0) | 0 (0.0) | 0 (0.0) |
| Stomatitis | 5 (29.4) | 1 (5.9) | 4 (23.5) | 0 (0.0) | 0 (0.0) | 0 (0.0) | 0 (0.0) | 0 (0.0) | 0 (0.0) | 0 (0.0) |
| Back pain | 4 (23.5) | 2 (11.8) | 1 (5.9) | 0 (0.0) | 1 (5.9) | 1 (9.1) | 0 (0.0) | 1 (9.1) | 0 (0.0) | 0 (0.0) |
| Nasopharyngitis | 4 (23.5) | 2 (11.8) | 2 (11.8) | 0 (0.0) | 0 (0.0) | 3 (27.3) | 2 (18.2) | 1 (9.1) | 0 (0.0) | 0 (0.0) |
| Neuropathy peripheral | 4 (23.5) | 2 (11.8) | 2 (11.8) | 0 (0.0) | 0 (0.0) | 0 (0.0) | 0 (0.0) | 0 (0.0) | 0 (0.0) | 0 (0.0) |
| Thrombocytopenia | 4 (23.5) | 0 (0.0) | 3 (17.6) | 1 (5.9) | 0 (0.0) | 0 (0.0) | 0 (0.0) | 0 (0.0) | 0 (0.0) | 0 (0.0) |
| Upper respiratory tract infection | 4 (23.5) | 1 (5.9) | 2 (11.8) | 1 (5.9) | 0 (0.0) | 1 (9.1) | 0 (0.0) | 1 (9.1) | 0 (0.0) | 0 (0.0) |
| Vomiting | 4 (23.5) | 1 (5.9) | 3 (17.6) | 0 (0.0) | 0 (0.0) | 2 (18.2) | 1 (9.1) | 0 (0.0) | 1 (9.1) | 0 (0.0) |
| Cough | 3 (17.6) | 3 (17.6) | 0 (0.0) | 0 (0.0) | 0 (0.0) | 1 (9.1) | 1 (9.1) | 0 (0.0) | 0 (0.0) | 0 (0.0) |
| Dizziness | 3 (17.6) | 3 (17.6) | 0 (0.0) | 0 (0.0) | 0 (0.0) | 0 (0.0) | 0 (0.0) | 0 (0.0) | 0 (0.0) | 0 (0.0) |
| Epistaxis | 3 (17.6) | 3 (17.6) | 0 (0.0) | 0 (0.0) | 0 (0.0) | 0 (0.0) | 0 (0.0) | 0 (0.0) | 0 (0.0) | 0 (0.0) |
| Headache | 3 (17.6) | 2 (11.8) | 1 (5.9) | 0 (0.0) | 0 (0.0) | 0 (0.0) | 0 (0.0) | 0 (0.0) | 0 (0.0) | 0 (0.0) |
| Hot flush | 3 (17.6) | 3 (17.6) | 0 (0.0) | 0 (0.0) | 0 (0.0) | 0 (0.0) | 0 (0.0) | 0 (0.0) | 0 (0.0) | 0 (0.0) |
| Influenza | 3 (17.6) | 2 (11.8) | 0 (0.0) | 1 (5.9) | 0 (0.0) | 0 (0.0) | 0 (0.0) | 0 (0.0) | 0 (0.0) | 0 (0.0) |
| Local swelling | 3 (17.6) | 3 (17.6) | 0 (0.0) | 0 (0.0) | 0 (0.0) | 0 (0.0) | 0 (0.0) | 0 (0.0) | 0 (0.0) | 0 (0.0) |
| Musculoskeletal pain | 3 (17.6) | 2 (11.8) | 0 (0.0) | 1 (5.9) | 0 (0.0) | 1 (9.1) | 0 (0.0) | 1 (9.1) | 0 (0.0) | 0 (0.0) |
| Pain in extremity | 3 (17.6) | 1 (5.9) | 2 (11.8) | 0 (0.0) | 0 (0.0) | 3 (27.3) | 2 (18.2) | 1 (9.1) | 0 (0.0) | 0 (0.0) |
| Rash | 3 (17.6) | 2 (11.8) | 1 (5.9) | 0 (0.0) | 0 (0.0) | 0 (0.0) | 0 (0.0) | 0 (0.0) | 0 (0.0) | 0 (0.0) |
| Urinary tract infection | 3 (17.6) | 0 (0.0) | 3 (17.6) | 0 (0.0) | 0 (0.0) | 0 (0.0) | 0 (0.0) | 0 (0.0) | 0 (0.0) | 0 (0.0) |
| Alanine aminotransferase increased | 2 (11.8) | 2 (11.8) | 0 (0.0) | 0 (0.0) | 0 (0.0) | 1 (9.1) | 0 (0.0) | 1 (9.1) | 0 (0.0) | 0 (0.0) |
| Aspartate aminotransferase increased | 2 (11.8) | 1 (5.9) | 1 (5.9) | 0 (0.0) | 0 (0.0) | 1 (9.1) | 0 (0.0) | 1 (9.1) | 0 (0.0) | 0 (0.0) |
| Blood alkaline phosphatase increased | 2 (11.8) | 1 (5.9) | 1 (5.9) | 0 (0.0) | 0 (0.0) | 0 (0.0) | 0 (0.0) | 0 (0.0) | 0 (0.0) | 0 (0.0) |
| Bone pain | 2 (11.8) | 1 (5.9) | 1 (5.9) | 0 (0.0) | 0 (0.0) | 1 (9.1) | 1 (9.1) | 0 (0.0) | 0 (0.0) | 0 (0.0) |
| Constipation | 2 (11.8) | 1 (5.9) | 1 (5.9) | 0 (0.0) | 0 (0.0) | 2 (18.2) | 0 (0.0) | 2 (18.2) | 0 (0.0) | 0 (0.0) |
| Dry skin | 2 (11.8) | 2 (11.8) | 0 (0.0) | 0 (0.0) | 0 (0.0) | 0 (0.0) | 0 (0.0) | 0 (0.0) | 0 (0.0) | 0 (0.0) |
| Dyspepsia | 2 (11.8) | 2 (11.8) | 0 (0.0) | 0 (0.0) | 0 (0.0) | 0 (0.0) | 0 (0.0) | 0 (0.0) | 0 (0.0) | 0 (0.0) |
| Erythema | 2 (11.8) | 2 (11.8) | 0 (0.0) | 0 (0.0) | 0 (0.0) | 0 (0.0) | 0 (0.0) | 0 (0.0) | 0 (0.0) | 0 (0.0) |
| Fall | 2 (11.8) | 1 (5.9) | 1 (5.9) | 0 (0.0) | 0 (0.0) | 1 (9.1) | 0 (0.0) | 1 (9.1) | 0 (0.0) | 0 (0.0) |
| Hypertension | 2 (11.8) | 0 (0.0) | 2 (11.8) | 0 (0.0) | 0 (0.0) | 0 (0.0) | 0 (0.0) | 0 (0.0) | 0 (0.0) | 0 (0.0) |
| Lymphoedema | 2 (11.8) | 2 (11.8) | 0 (0.0) | 0 (0.0) | 0 (0.0) | 0 (0.0) | 0 (0.0) | 0 (0.0) | 0 (0.0) | 0 (0.0) |
| Pyrexia | 2 (11.8) | 2 (11.8) | 0 (0.0) | 0 (0.0) | 0 (0.0) | 0 (0.0) | 0 (0.0) | 0 (0.0) | 0 (0.0) | 0 (0.0) |
| Toothache | 2 (11.8) | 1 (5.9) | 1 (5.9) | 0 (0.0) | 0 (0.0) | 1 (9.1) | 1 (9.1) | 0 (0.0) | 0 (0.0) | 0 (0.0) |

Table S10: Most common all-cause adverse events that occurred in at least 10% of patients with visceral metastases in the palbociclib + letrozole arm of the safety population

|  | **Palbociclib + Letrozole**  **n = 37** | | | | | **Letrozole**  **n = 43** | | | | |
| --- | --- | --- | --- | --- | --- | --- | --- | --- | --- | --- |
| **Preferred Term** | **All Grades**  **n (%)** | **Grade 1**  **n (%)** | **Grade 2**  **n (%)** | **Grade 3**  **n (%)** | **Grade 4**  **n (%)** | **All Grades**  **n (%)** | **Grade 1**  **n (%)** | **Grade 2**  **n (%)** | **Grade 3**  **n (%)** | **Grade 4**  **n (%)** |
| Any AEs | 37 (100.0) | 1 (2.7) | 9 (24.3) | 20 (54.1) | 6 (16.2) | 36 (83.7) | 9 (20.9) | 15 (34.9) | 12 (27.9) | 0 (0.0) |
| Neutropenia | 24 (64.9) | 1 (2.7) | 6 (16.2) | 16 (43.2) | 1 (2.7) | 2 (4.7) | 1 (2.3) | 0 (0.0) | 1 (2.3) | 0 (0.0) |
| Leukopenia | 17 (45.9) | 4 (10.8) | 7 (18.9) | 6 (16.2) | 0 (0.0) | 0 (0.0) | 0 (0.0 | 0 (0.0) | 0 (0.0) | 0 (0.0) |
| Fatigue | 15 (40.5) | 7 (18.9) | 6 (16.2) | 0 (0.0) | 2 (5.4) | 10 (23.3) | 5 (11.6) | 4 (9.3) | 1 (2.3) | 0 (0.0) |
| Anemia | 14 (37.8) | 0 (0.0) | 12 (32.4) | 2 (5.4) | 0 (0.0) | 3 (7.0) | 0 (0.0) | 2 (4.7) | 1 (2.3) | 0 (0.0) |
| Decreased appetite | 10 (27.0) | 8 (21.6) | 1 (2.7) | 1 (2.7) | 0 (0.0) | 4 (9.3) | 3 (7.0) | 1 (2.3) | 0 (0.0) | 0 (0.0) |
| Arthralgia | 9 (24.3) | 5 (13.5) | 4 (10.8) | 0 (0.0) | 0 (0.0) | 6 (14.0) | 3 (7.0) | 1 (2.3) | 2 (4.7) | 0 (0.0) |
| Dyspnea | 9 (24.3) | 4 (10.8) | 3 (8.1) | 2 (5.4) | 0 (0.0) | 4 (9.3) | 1 (2.3) | 2 (4.7) | 1 (2.3) | 0 (0.0) |
| Nausea | 8 (21.6) | 6 (16.2) | 1 (2.7) | 1 (2.7) | 0 (0.0) | 5 (11.6) | 3 (7.0) | 2 (4.7) | 0 (0.0) | 0 (0.0) |
| Alopecia | 7 (18.9) | 6 (16.2) | 1 (2.7) | 0 (0.0) | 0 (0.0) | 1 (2.3) | 1 (2.3) | 0 (0.0) | 0 (0.0) | 0 (0.0) |
| Thrombocytopenia | 7 (18.9) | 4 (10.8) | 2 (5.4) | 1 (2.7) | 0 (0.0) | 1 (2.3) | 1 (2.3) | 0 (0.0) | 0 (0.0) | 0 (0.0) |
| Asthenia | 6 (16.2) | 3 (8.1) | 3 (8.1) | 0 (0.0) | 0 (0.0) | 3 (7.0) | 3 (7.0) | 0 (0.0) | 0 (0.0) | 0 (0.0) |
| Abdominal pain | 5 (13.5) | 4 (10.8) | 0 (0.0) | 1 (2.7) | 0 (0.0) | 3 (7.0) | 2 (4.7) | 1 (2.3) | 0 (0.0) | 0 (0.0) |
| Headache | 5 (13.5) | 4 (10.8) | 1 (2.7) | 0 (0.0) | 0 (0.0) | 4 (9.3) | 2 (4.7) | 2 (4.7) | 0 (0.0) | 0 (0.0) |
| Hot flush | 5 (13.5) | 5 (13.5) | 0 (0.0) | 0 (0.0) | 0 (0.0) | 5 (11.6) | 4 (9.3) | 1 (2.3) | 0 (0.0) | 0 (0.0) |
| Nasopharyngitis | 5 (13.5) | 5 (13.5) | 0 (0.0) | 0 (0.0) | 0 (0.0) | 4 (9.3) | 3 (7.0) | 1 (2.3) | 0 (0.0) | 0 (0.0) |
| Pain in extremity | 5 (13.5) | 4 (10.8) | 1 (2.7) | 0 (0.0) | 0 (0.0) | 1 (2.3) | 1 (2.3) | 0 (0.0) | 0 (0.0) | 0 (0.0) |
| Vomiting | 5 (13.5) | 5 (13.5) | 0 (0.0) | 0 (0.0) | 0 (0.0) | 1 (2.3) | 1 (2.3) | 0 (0.0) | 0 (0.0) | 0 (0.0) |
| Blood alkaline phosphatase increased | 4 (10.8) | 1 (2.7) | 3 (8.1) | 0 (0.0) | 0 (0.0) | 2 (4.7) | 2 (4.7) | 0 (0.0) | 0 (0.0) | 0 (0.0) |
| Blood creatinine increased | 4 (10.8) | 2 (5.4) | 2 (5.4) | 0 (0.0) | 0 (0.0) | 2 (4.7) | 1 (2.3) | 1 (2.3) | 0 (0.0) | 0 (0.0) |
| Bone pain | 4 (10.8) | 0 (0.0) | 3 (8.1) | 0 (0.0) | 1 (2.7) | 2 (4.7) | 1 (2.3) | 1 (2.3) | 0 (0.0) | 0 (0.0) |
| Diarrhea | 4 (10.8) | 1 (2.7) | 1 (2.7) | 2 (5.4) | 0 (0.0) | 4 (9.3) | 2 (4.7) | 2 (4.7) | 0 (0.0) | 0 (0.0) |
| Dysgeusia | 4 (10.8) | 4 (10.8) | 0 (0.0) | 0 (0.0) | 0 (0.0) | 0 (0.0) | 0 (0.0) | 0 (0.0) | 0 (0.0) | 0 (0.0) |
| Dysuria | 4 (10.8) | 3 (8.1) | 1 (2.7) | 0 (0.0) | 0 (0.0) | 2 (4.7) | 1 (2.3) | 1 (2.3) | 0 (0.0) | 0 (0.0) |
| Musculoskeletal pain | 4 (10.8) | 2 (5.4) | 2 (5.4) | 0 (0.0) | 0 (0.0) | 2 (4.7) | 1 (2.3) | 1 (2.3) | 0 (0.0) | 0 (0.0) |

Table S11: Most common all-cause adverse events that occurred in at least 10% of patients with other metastases (bone with other non-visceral sites or other disease sites alone) in the palbociclib + letrozole arm of the safety population

|  | **Palbociclib + Letrozole**  **n = 29** | | | | | **Letrozole**  **n = 23** | | | | |
| --- | --- | --- | --- | --- | --- | --- | --- | --- | --- | --- |
| **Preferred Term** | **All Grades**  **n (%)** | **Grade 1**  **n (%)** | **Grade 2**  **n (%)** | **Grade 3**  **n (%)** | **Grade 4**  **n (%)** | **All Grades**  **n (%)** | **Grade 1**  **n (%)** | **Grade 2**  **n (%)** | **Grade 3**  **n (%)** | **Grade 4**  **n (%)** |
| Any AEs | 29 (100.0) | 0 (0.0) | 7 (24.1) | 17 (58.6) | 5 (17.2) | 19 (82.6) | 6 (26.1) | 11 (47.8) | 2 (8.7) | 0 (0.0) |
| Neutropenia | 24 (82.8) | 1 (3.4) | 7 (24.1) | 13 (44.8) | 3 (10.3) | 2 (8.7) | 0 (0.0) | 2 (8.7) | 0 (0.0) | 0 (0.0) |
| Leukopenia | 12 (41.4) | 1 (3.4) | 5 (17.2) | 6 (20.7) | 0 (0.0) | 2 (8.7) | 0 (0.0) | 2 (8.7) | 0 (0.0) | 0 (0.0) |
| Anemia | 9 (31.0) | 4 (13.8) | 4 (13.8) | 1 (3.4) | 0 (0.0) | 1 (4.3) | 0 (0.0) | 1 (4.3) | 0 (0.0) | 0 (0.0) |
| Fatigue | 9 (31.0) | 2 (6.9) | 6 (20.7) | 1 (3.4) | 0 (0.0) | 5 (21.7) | 4 (17.4) | 1 (4.3) | 0 (0.0) | 0 (0.0) |
| Hot flush | 9 (31.0) | 8 (27.6) | 1 (3.4) | 0 (0.0) | 0 (0.0) | 4 (17.4) | 3 (13.0) | 1 (4.3) | 0 (0.0) | 0 (0.0) |
| Diarrhea | 8 (27.6) | 4 (13.8) | 3 (10.3) | 1 (3.4) | 0 (0.0) | 3 (13.0) | 2 (8.7) | 1 (4.3) | 0 (0.0) | 0 (0.0) |
| Alopecia | 6 (20.7) | 6 (20.7) | 0 (0.0) | 0 (0.0) | 0 (0.0) | 0 (0.0) | 0 (0.0) | 0 (0.0) | 0 (0.0) | 0 (0.0) |
| Constipation | 6 (20.7) | 5 (17.2) | 1 (3.4) | 0 (0.0) | 0 (0.0) | 2 (8.7) | 2 (8.7) | 0 (0.0) | 0 (0.0) | 0 (0.0) |
| Nausea | 6 (20.7) | 4 (13.8) | 1 (3.4) | 1 (3.4) | 0 (0.0) | 3 (13.0) | 2 (8.7) | 1 (4.3) | 0 (0.0) | 0 (0.0) |
| Arthralgia | 5 (17.2) | 2 (6.9) | 3 (10.3) | 0 (0.0) | 0 (0.0) | 3 (13.0) | 2 (8.7) | 1 (4.3) | 0 (0.0) | 0 (0.0) |
| Back pain | 5 (17.2) | 4 (13.8) | 1 (3.4) | 0 (0.0) | 0 (0.0) | 4 (17.4) | 1 (4.3) | 3 (13.0) | 0 (0.0) | 0 (0.0) |
| Cough | 5 (17.2) | 2 (6.9) | 3 (10.3) | 0 (0.0) | 0 (0.0) | 4 (17.4) | 4 (17.4) | 0 (0.0) | 0 (0.0) | 0 (0.0) |
| Asthenia | 4 (13.8) | 3 (10.3) | 0 (0.0) | 1 (3.4) | 0 (0.0) | 0 (0.0) | 0 (0.0) | 0 (0.0) | 0 (0.0) | 0 (0.0) |
| Bone pain | 4 (13.8) | 2 (6.9) | 1 (3.4) | 1 (3.4) | 0 (0.0) | 0 (0.0) | 0 (0.0) | 0 (0.0) | 0 (0.0) | 0 (0.0) |
| Epistaxis | 4 (13.8) | 4 (13.8) | 0 (0.0) | 0 (0.0) | 0 (0.0) | 0 (0.0) | 0 (0.0) | 0 (0.0) | 0 (0.0) | 0 (0.0) |
| Headache | 4 (13.8) | 2 (6.9) | 2 (6.9) | 0 (0.0) | 0 (0.0) | 4 (17.4) | 2 (8.7) | 2 (8.7) | 0 (0.0) | 0 (0.0) |
| Influenza | 4 (13.8) | 1 (3.4) | 3 (10.3) | 0 (0.0) | 0 (0.0) | 0 (0.0) | 0 (0.0) | 0 (0.0) | 0 (0.0) | 0 (0.0) |
| Nasopharyngitis | 4 (13.8) | 3 (10.3) | 1 (3.4) | 0 (0.0) | 0 (0.0) | 1 (4.3) | 0 (0.0) | 1 (4.3) | 0 (0.0) | 0 (0.0) |
| Dizziness | 3 (10.3) | 2 (6.9) | 1 (3.4) | 0 (0.0) | 0 (0.0) | 2 (8.7) | 2 (8.7) | 0 (0.0) | 0 (0.0) | 0 (0.0) |
| Dyspepsia | 3 (10.3) | 2 (6.9) | 1 (3.4) | 0 (0.0) | 0 (0.0) | 1 (4.3) | 1 (4.3) | 0 (0.0) | 0 (0.0) | 0 (0.0) |
| Dyspnea | 3 (10.3) | 3 (10.3) | 0 (0.0) | 0 (0.0) | 0 (0.0) | 2 (8.7) | 2 (8.7) | 0 (0.0) | 0 (0.0) | 0 (0.0) |
| Gingival pain | 3 (10.3) | 1 (3.4) | 2 (6.9) | 0 (0.0) | 0 (0.0) | 0 (0.0) | 0 (0.0) | 0 (0.0) | 0 (0.0) | 0 (0.0) |
| Insomnia | 3 (10.3) | 2 (6.9) | 1 (3.4) | 0 (0.0) | 0 (0.0) | 3 (13.0) | 2 (8.7) | 1 (4.3) | 0 (0.0) | 0 (0.0) |
| Joint stiffness | 3 (10.3) | 2 (6.9) | 0 (0.0) | 1 (3.4) | 0 (0.0) | 1 (4.3) | 1 (4.3) | 0 (0.0) | 0 (0.0) | 0 (0.0) |
| Mood altered | 3 (10.3) | 2 (6.9) | 1 (3.4) | 0 (0.0) | 0 (0.0) | 1 (4.3) | 1 (4.3) | 0 (0.0) | 0 (0.0) | 0 (0.0) |
| Mucosal inflammation | 3 (10.3) | 2 (6.9) | 1 (3.4) | 0 (0.0) | 0 (0.0) | 0 (0.0) | 0 (0.0) | 0 (0.0) | 0 (0.0) | 0 (0.0) |
| Edema peripheral | 3 (10.3) | 2 (6.9) | 1 (3.4) | 0 (0.0) | 0 (0.0) | 3 (13.0) | 3 (13.0) | 0 (0.0) | 0 (0.0) | 0 (0.0) |
| Pain | 3 (10.3) | 1 (3.4) | 1 (3.4) | 1 (3.4) | 0 (0.0) | 1 (4.3) | 1 (4.3) | 0 (0.0) | 0 (0.0) | 0 (0.0) |
| Pyrexia | 3 (10.3) | 3 (10.3) | 0 (0.0) | 0 (0.0) | 0 (0.0) | 0 (0.0) | 0 (0.0) | 0 (0.0) | 0 (0.0) | 0 (0.0) |
| Thrombocytopenia | 3 (10.3) | 3 (10.3) | 0 (0.0) | 0 (0.0) | 0 (0.0) | 0 (0.0) | 0 (0.0) | 0 (0.0) | 0 (0.0) | 0 (0.0) |
| Toothache | 3 (10.3) | 2 (6.9) | 1 (3.4) | 0 (0.0) | 0 (0.0) | 0 (0.0) | 0 (0.0) | 0 (0.0) | 0 (0.0) | 0 (0.0) |
| Upper respiratory tract infection | 3 (10.3) | 2 (6.9) | 1 (3.4) | 0 (0.0) | 0 (0.0) | 0 (0.0) | 0 (0.0) | 0 (0.0) | 0 (0.0) | 0 (0.0) |
| Urinary tract infection | 3 (10.3) | 0 (0.0) | 3 (10.3) | 0 (0.0) | 0 (0.0) | 2 (8.7) | 1 (4.3) | 1 (4.3) | 0 (0.0) | 0 (0.0) |
| Vomiting | 3 (10.3) | 2 (6.9) | 1 (3.4) | 0 (0.0) | 0 (0.0) | 0 (0.0) | 0 (0.0) | 0 (0.0) | 0 (0.0) | 0 (0.0) |
